# LINKER: Learning Interactions between Functional Groups and Residues with Chemical Knowledge‑Enhanced Reasoning and Explainability

**DOI:** 10.1021/acs.jcim.6c00527

**Published:** 2026-07-16

**Authors:** Phuc Pham, Viet Thanh Duy Nguyen, Kevin Song, Jake Chen, Truong-Son Hy

**Affiliations:** † Department of Computer Science, 200297 The University of Alabama at Birmingham, Birmingham, Alabama 35294, United States; ‡ Department of Biomedical Engineering, 9968 The University of Alabama at Birmingham, Birmingham, Alabama 35294, United States; § Department of Biomedical Informatics and Data Science, The University of Alabama at Birmingham, Birmingham, Alabama 35294, United States

## Abstract

Accurate identification of interactions between protein residues and ligand functional groups is critical for understanding molecular recognition and guiding rational drug design. Existing deep learning approaches for protein–ligand interpretability typically rely on three-dimensional structural input or distance-based contact labels, which limit both their applicability and biological relevance. Here, we present LINKER, the first sequence-based model to predict residue-functional group interactions according to biologically defined interaction types, using only a protein sequence and the SMILES representation of the ligand. LINKER is trained via structure-supervised interaction learning, in which interaction labels are derived from three-dimensional protein–ligand complexes through functional group-based motif extraction. By representing ligands as ensembles of functional groups, the model emphasizes chemically meaningful substructures rather than mere spatial proximity. Importantly, LINKER requires only sequence-level input at inference, enabling large-scale applications in contexts where structural data are unavailable. Extensive experiments demonstrate that LINKER consistently outperforms established baselines, highlighting the utility of functional group abstractions and structure-based supervision for interpretable protein–ligand interaction prediction. Our source code is publicly available at: https://github.com/HySonLab/LINKER/.

## Graphical abstract

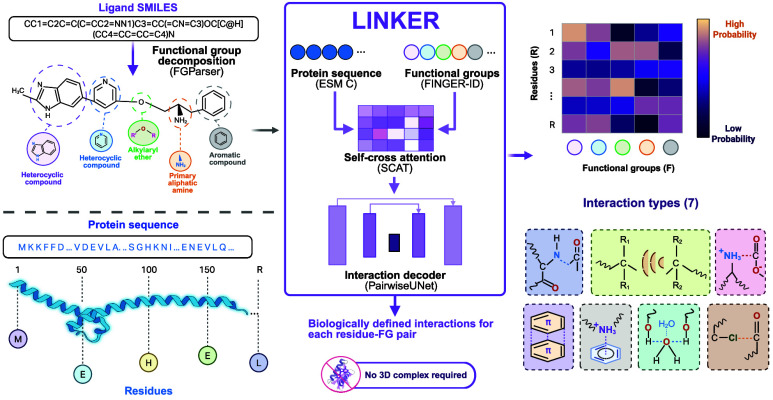

## 1 Introduction

Protein–ligand interactions underpin virtually all processes in chemical biology and pharmacology. Understanding how a ligand engages with its target protein, particularly which functional groups form what types of interactions with specific residues, is critical for elucidating molecular recognition mechanisms, rationalizing structure–activity relationships, and guiding lead optimization in drug discovery. In existing work, obtaining detailed protein–ligand interaction maps almost always requires access to a three-dimensional complex structure. However, such complexes are not always experimentally available and, when they are missing, researchers typically resort to molecular docking between a protein structure and a ligand using simulation tools such as AutoDock Vina,^1^ followed by postprocessing with tools such as PLIP^2^ to extract interaction types. This process can be computationally expensive and particularly time-consuming, especially in the case of blind docking, in which the binding pocket is unknown and the search space is large.^3^

To the best of our knowledge, no existing method directly predicts biologically defined interactions between protein residues and ligand functional groups from sequence-level input alone. Some sequence-based binding affinity models introduce structure-supervised attention to generate residue-ligand interaction maps as an auxiliary signal.^4–7^ However, because these maps are not explicitly supervised as a primary objective, their interpretability and accuracy are often limited. Other approaches focus on identifying binding residues from protein sequences.^8–10^ Still, they typically ignore the ligand’s chemical context, particularly the specific functional groups involved, thereby overlooking key determinants of biochemical specificity. Critically, these models do not incorporate functional group abstractions or biologically meaningful interaction types, which limits their ability to capture interpretable and chemically grounded interaction patterns.

In this study, we introduce LINKER, a novel sequence-based framework capable of directly predicting interactions between protein residues and ligand functional groups using only protein sequences and ligand SMILES strings as inputs. The model is trained using guidance from determined protein–ligand complexes, where interaction labels are extracted by PLIP after ligands are decomposed into chemically relevant functional groups. This strategy enables LINKER to focus on the substructures that drive molecular recognition and to predict biologically meaningful interaction types, such as hydrogen bonds, π-stacking, and salt bridges, rather than relying solely on interatomic distances. Notably, LINKER performs predictions entirely at the sequence level during inference, allowing large-scale application to protein–ligand pairs without available structural data, while retaining chemical interpretability aligned with medicinal chemistry reasoning.

## 2 Problem Formulation

We cast residue-functional group interaction prediction as a multilabel classification task. Given a protein–ligand pair, the objective is to estimate, for every protein residue and each ligand functional group (FG), independent probabilities over seven biologically defined interaction types: hydrogen bonds, hydrophobic contacts, π-stacking, π-cation interactions, salt bridges, water bridges, and halogen bonds. Formally, let **T** denote the protein amino acid sequence, comprising *R* residues, and let **D** denote the ligand SMILES sequence, decomposed into *F* functional groups. Our model, LINKER, learns the following mapping

1 $$f_{\mathrm{LINKER}}:\left(T,D\right)\to P\in {\left[0,1\right]}^{R\times F\times 7}$$

where **P**_*r*,*f*,*k*_ denotes the likelihood that residue *r* and functional group *f* are involved in the interaction type *k*, with *k* = 1,..., 7. Unlike contact maps that only indicate spatial proximity, in which residues may be close but not engaged in any biochemical interaction, LINKER provides direct supervision over interaction types, enabling chemically grounded reasoning and improved interpretability.

## 3 Method

We propose LINKER, a sequence-based framework for predicting biologically defined residue-functional group interaction types from protein sequences and ligand SMILES. As illustrated in Figure 1, LINKER is organized into three main components: (i) a protein representation implemented by the *Protein Language Model* (module 1), (ii) a ligand representation consisting of *FGParser* (module 2) and *FINGER-ID* (module 3), and (iii) an interaction modeling that integrates protein and ligand features using *SCAT* (module 4) and *PairwiseUNet* (module 5). Together, these components encompass five functional modules that produce interpretable residue-functional group interaction maps directly from sequence-level inputs.

**Figure 1:**
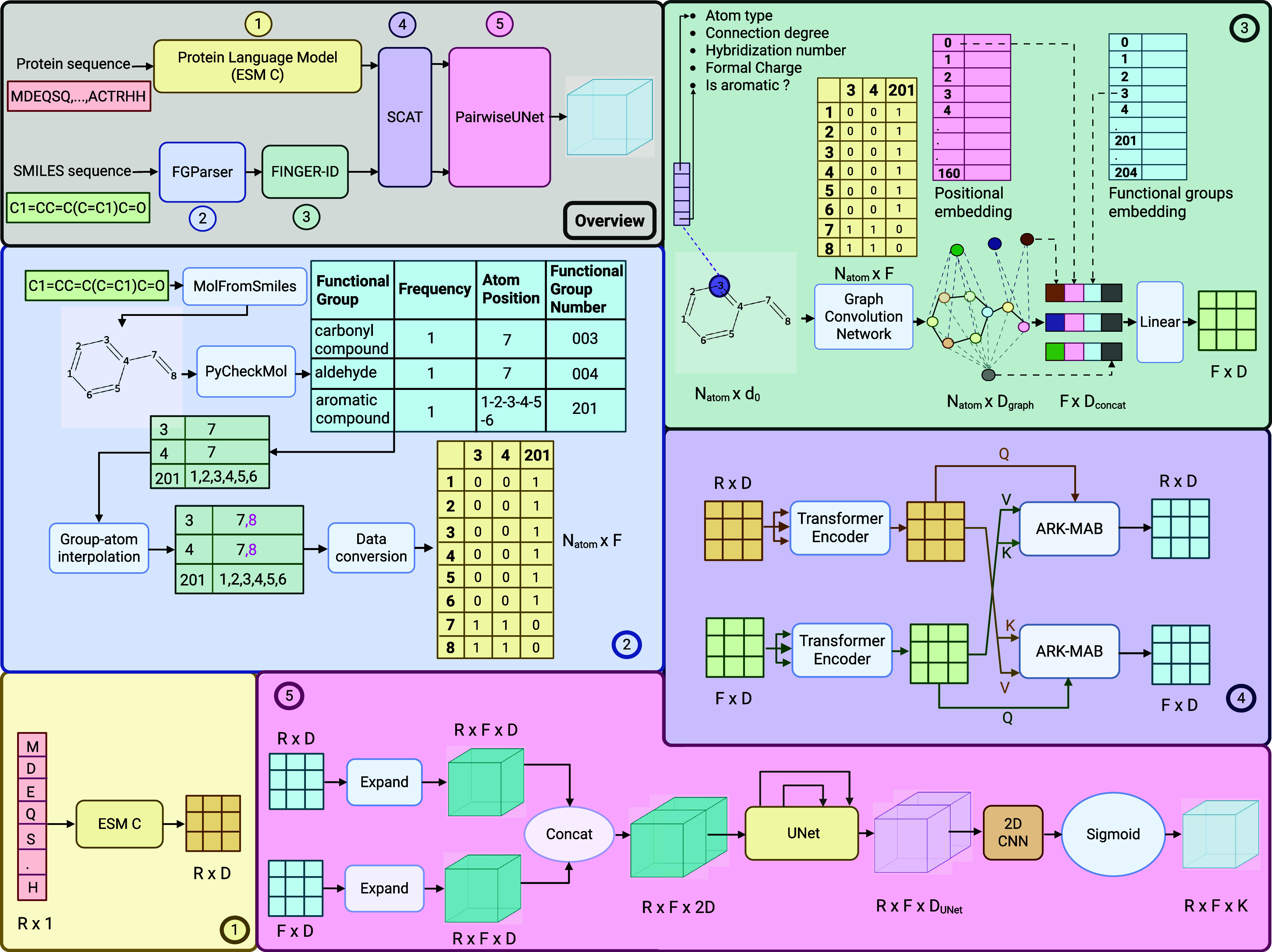
Architecture of LINKER. LINKER predicts residue-functional group interaction types from protein sequences and ligand SMILES. (1) A protein language model (ESM C) encodes residue embeddings. (2) FGParser extracts ligand functional groups. (3) FINGER-ID generates context-aware group embeddings. (4) SCAT integrates protein and ligand representations via self- and cross-attention. (5) PairwiseUNet outputs interaction probabilities for each residue-functional group pair.

### 3.1 Protein Representation

The protein sequence is encoded using the *ESM C* ^11^ *protein language model*, which is optimized to capture biologically meaningful representations of proteins. Unlike generative models such as ESM-3,^12^ which focus on controllable sequence generation, ESM C specializes in representation learning and supports multichain inputs, making it more versatile than prior models^13^ for capturing complex protein–ligand interactions without requiring structural data.

Formally, let the protein target be represented by a FASTA sequence **T** = (*t*_1_, *t*_2_,..., *t*_*R*_), where *R* is the sequence length and each residue $t_{r}\in A$ for *r* = 1,..., *R*. Here, A denotes the amino acid alphabet. The sequence is first tokenized and then processed by the ESM C encoder, yielding residue-level embeddings

2 $$H_{p}=f_{\theta }\left(T\right):A^{R}\to R^{R\times D},,H_{p}=\left[\begin{array}{l} h_{1}^{T}\\ \vdots \\ h_{R}^{T} \end{array}\right]\in R^{R\times D}$$

Each embedding **h**_*r*_ captures both the local biochemical properties of residue *t*_*r*_ and its long-range dependencies within the full protein sequence. These contextualized features enable our framework to utilize structural and evolutionary information learned from large-scale protein corpora without requiring explicit three-dimensional structures. In our implementation, we use the ESM C (300 M parameter) variant with hidden size *D* = 960, resulting in an output matrix $H_{p}\in R^{R\times 960}$ .

### 3.2 Ligand Representation

Inspired by the development of multiscale models for complex chemical systems,^14^ we adopted a higher-level ligand representation based on functional groups rather than individual atoms. This abstraction aligns more naturally with protein–ligand interactions, which are typically governed by chemically meaningful substructures in the ligand and complementary residues in the protein. In contrast, traditional fingerprints such as Morgan/ECFP4 ^15,16^ represent atom-centered environments without global context, often yielding partial motifs that lack semantic interpretability and cannot distinguish multiple instances of the same group. These limitations motivate our use of explicit functional group representations, which can provide both chemical interpretability and biological relevance.

To realize this, we introduce two complementary modules: the *Functional Group Parser* (*FGParser*), which deterministically maps ligand SMILES into an atom-group representation matrix, and the *Functional Group and Positional Embedding for Molecular Identification* (*FINGER-ID*), which constructs hierarchical, context-aware embeddings of functional groups.

#### 3.2.1 Functional Group Parser (FGParser)

The FGParser module decomposes a ligand into its constituent functional groups and generates an atom-group representation matrix for downstream processing. The procedure comprises two stages: atom-group mapping and group-atom interpolation.

##### 3.2.1.1 Atom-Group Mapping

Given a ligand SMILES sequence, denoted **D**, FGParser first uses RDKit^17^ to convert the SMILES into a Mol object, which encodes the atom and bond information in a structured format suitable for cheminformatics operations. Functional groups are then identified using PyCheckMol^18^ (see Supporting Information A for details), which applies a curated set of predefined chemical patterns to detect chemically significant substructures. Each detected functional group is assigned a unique identifier, and all atoms belonging to that group are tagged with the corresponding ID. This process produces an initial mapping of functional groups to their member atoms, preserving explicit atom–group associations for downstream processing.

##### 3.2.1.2 Group-Atom Interpolation

Some atoms may not be assigned to any canonical functional group during the initial stage. To ensure complete coverage, these atoms are allocated to the nearest functional group based on index distance along the molecular graph. Ties between equidistant groups are resolved by the ascending group ID.

After this assignment step, we construct a binary matrix: $M\in R^{N_{\mathrm{atom}}\times F}$ defined as

3 $$M_{i,j}=\{\begin{array}{ll} 1 & \mathrm{if}\;\mathrm{atom}\,\;i\;belongs\,\;to\,\;\mathrm{or}\,\;\mathrm{is}\,\;\mathrm{assigned}\,\;\mathrm{to}\,\;\mathrm{FG}j,\\ 0 & \mathrm{otherwise} \end{array}$$

This conversion preserves atom-level resolution while incorporating functional group membership, enabling downstream modules to utilize both structural and chemical information.

#### 3.2.2 Functional Group and Positional Embedding for Molecular Identification (FINGER-ID)

The FINGER-ID module encodes ligands at the functional-group level while preserving atom-level structural information for interaction modeling. This design is motivated by the observation that protein–ligand recognition is often governed by chemically coherent substructures, such as hydrogen-bond donors and acceptors, aromatic systems, charged groups, and hydrophobic moieties, rather than by isolated atoms alone. FINGER-ID therefore organizes atomic features into hierarchical, context-aware functional-group embeddings that integrate local connectivity, functional-group identity, positional information, and global molecular context. Unlike conventional fingerprints such as Morgan fingerprints, which represent atom-centered environments as fixed descriptors, FINGER-ID generates explicit embeddings for individual functional groups and distinguishes repeated groups in different structural contexts. These representations provide a chemically interpretable ligand abstraction for residue-functional group interaction reasoning and downstream molecular prediction tasks.

##### 3.2.2.1 Atomic-Scale (Atomic Property) Initialization

We represent a molecule as a graph *G* = (*V*, *E*) with |*V*| = *N* atoms. Each atom *a* ∈ *V* is associated with a feature vector $x_{a}\in R^{d_{0}}$ that encodes its atomic properties. In our implementation, we set *d*_0_ = 5, which corresponds to the type, degree, hybridization, formal charge, and aromaticity of the atom. The node features are collected into a matrix

4 $$X=\left[x_{1}^{T};...;x_{N}^{T}\right]\in R^{N\times d_{0}}$$

The edge set *E* is defined by chemical bonds that capture molecular connectivity.

##### 3.2.2.2 Local-Scale (Neighborhood) Encoding

We encode the graph topology and node features with a graph convolutional network (GCN) to obtain latent atom embeddings

5 $$Z=\mathrm{GCN}\left(G,X\right)\in R^{N\times D_{\mathrm{graph}}}$$

where the *a*th row **z**_*a*_ represents the embedding of atom *a*. This stage captures local-scale connectivity patterns within atomic neighborhoods based solely on adjacency information.

##### 3.2.2.3 Intermediate-Scale (Functional Group) Embedding

Each functional group *g* is represented by aggregating the embeddings of its constituent atoms according to the atom-to-group mapping M(g)⊆V obtained from FGParser

6 $$z_{g}^{\mathrm{inter}}=\mathrm{AGG}\left\{z_{a}|a\in M\left(g\right)\right\},g=1,...,F$$

where AGG denotes mean pooling. The group-level representation is then concatenated with a learnable group-type embedding **e**_*g*_^FG^ and a positional embedding **e**_*g*_^pos^ to distinguish multiple occurrences of the same group.

##### 3.2.2.4 Global-Scale (Molecular) Embedding

A global embedding is derived by applying a READOUT operation over all atom embeddings

7 $$z^{\mathrm{global}}=\mathrm{READOUT}\left(Z\right)$$

Finally, each functional group embedding is augmented with a global molecular context.

8 $$h_{g}=\left[z_{g}^{\mathrm{inter}}∥e_{g}^{\mathrm{FG}}∥e_{g}^{\mathrm{pos}}∥z^{\mathrm{global}}\right]$$

yielding a multiscale representation that integrates atomic features, local-scale neighborhood information, functional group semantics, and global molecular structure.

##### 3.2.2.5 Final Projection

All enriched group embeddings are stacked and linearly projected to obtain the final ligand representation:

9 $$H=\left[h_{1};...;h_{F}\right]\in R^{F\times D_{\mathrm{concat}}},H_{l}=\mathrm{HW}+b\in R^{F\times D}$$

By integrating atomic, local, intermediate, and global scale information, FINGER-ID produces position-aware, chemically interpretable embeddings that can differentiate identical functional groups in distinct chemical environments, thereby enhancing downstream binding and molecular property prediction.

To evaluate whether the functional-group abstraction provides a useful ligand representation, we performed an end-to-end ligand-representation ablation study on the Davis^19^ benchmark. Atom-level and functional-group-level variants were compared under an identical architecture and training protocol, such that the ligand representation was the only component varied. Across all evaluated GCN depths, the functional-group representation consistently outperformed the atom-level variant, indicating that the observed improvement is attributable to the representation choice rather than to a specific depth configuration. These results provide empirical support for the use of FINGER-ID as a chemically grounded ligand encoder; full experimental details are provided in Supporting Information D.

### 3.3 Interaction Modeling

To jointly model intra- and intermolecular dependencies and enable interpretable prediction of residue-functional group interactions, LINKER adopts an interaction modeling framework composed of two complementary modules: the *Selfand Cross Attention Transformer* (*SCAT*) and the *PairwiseUNet*. SCAT learns rich, sequence-derived contextual representations for proteins and ligands via self- and cross-attention mechanisms, capturing long-range dependencies within each modality as well as fine-grained coupling across modalities. Leveraging these contextualized representations, PairwiseUNet explicitly decodes residue-functional group interaction probability maps, providing an interpretable characterization of molecular recognition.

#### 3.3.1 Self and Cross Attention Transformer (SCAT)

To capture both intra- and intermolecular dependencies, we introduce the SCAT module. Let $H_{p}\in R^{R\times D}$ denote the protein residue embeddings obtained from ESM C, and $H_{l}\in R^{F\times D}$ denote the functional group embeddings of the ligand produced by FINGER-ID.

##### 3.3.1.1 Self-Attention

We utilize a self-attention mechanism to further encode the residue and functional-group embeddings

10 $$H_{p}^{\prime }={\mathrm{SA}}_{p}\left(H_{p}\right)$$

11 $$H_{l}^{\prime }={\mathrm{SA}}_{l}\left(H_{l}\right)$$

where SA_*p*_ and SA_*l*_ are transformer encoder blocks with self-attention applied within each modality.

##### 3.3.1.2 Cross-Attention

Furthermore, instead of employing standard cross-attention, we adopt the proposed ARK-MAB^8^ for protein–ligand integration, which explicitly reflects the biological reality that only a subset of protein residues participates in noncovalent interactions with the ligand, and similarly, only a subset of ligand substructures engages with the protein. To account for noninteracting elements in both molecules, ARK-MAB introduces a trainable pseudosubstructure embedding for nonbinding residues and a pseudoresidue embedding for nonbinding ligand substructures, each acting as an attention sink. Attention from noninteracting residues is directed toward the pseudosubstructure, while attention from noninteracting ligand substructures is directed toward the pseudoresidue, effectively suppressing spurious interactions in both directions. For interacting residues and substructures, attention is selectively distributed to the true partner embeddings, allowing meaningful exchange of interaction signals.

Mathematically, the bidirectional cross-attention can be expressed as

12 $$H_{p}^{\prime \prime }=\mathrm{ARK}-{\mathrm{MAB}}_{p\leftarrow l}\left(H_{p}^{\prime },H\prime_{l}\right)$$

13 $$H_{l}^{\prime \prime }=\mathrm{ARK}-{\mathrm{MAB}}_{l\leftarrow p}\left(H_{l}^{\prime },H_{p}^{\prime }\right)$$

where ARK-MAB_*p*←*l*_ attends from protein residues (queries) to ligand functional groups (keys/values), and ARK-MAB_*l*←*p*_ performs the reverse. The resulting outputs **H**_*p*_^″^ and **H**_*l*_^″^ incorporate intramolecular context from prior self-attention layers as well as cross-molecular interaction information, producing residue and substructure embeddings suitable for downstream modeling of protein–ligand interactions.

#### 3.3.2 PairwiseUNet

The PairwiseUNet module takes the enriched protein and ligand representations from SCAT, $H_{p}^{\prime \prime }\in R^{R\times D}$ and $H_{l}^{\prime \prime }\in R^{F\times D}$ , and transforms them into a structured pairwise feature tensor for interaction prediction.

##### 3.3.2.1 Pairwise Tensor Construction

Protein embeddings are transmitted along the functional group dimension to form $E_{p}\in R^{R\times F\times D}$ , while ligand embeddings are transmitted along the residue dimension to form $E_{l}\in R^{R\times F\times D}$ . These tensors are concatenated along the feature dimension, producing a joint representation

14 $$Z=\mathrm{Concat}\left(E_{p},E_{l}\right)\in R^{R\times F\times 2D}$$

##### 3.3.2.2 2D U-Net Processing

The combined tensor **Z** is processed by a 2D U-Net, which models both local and long-range spatial dependencies across the residue-functional group grid

15 $$U=\mathrm{UNet}\left(Z\right)\in R^{R\times F\times D_{\mathrm{unet}}}$$

##### 3.3.2.3 Interaction Type Prediction

Finally, a stack of 2D convolutional layers^20^ maps the pairwise feature tensor **U** to interaction-type probabilities

16 $$P=\mathrm{Sigmoid}\left(\mathrm{Conv}2D\left(U\right)\right)\in {\left[0,1\right]}^{R\times F\times K}$$

where *K* = 7 corresponds to biologically defined interaction types (e.g., hydrogen bonds, π-stacking, salt bridges). This produces an interpretable interaction map that can be directly derived from sequence-level input, with each entry **P**_*r*,*f*,*k*_ representing the independent probability of residue *r* and functional group *f* participating in the interaction type *k*, rather than a mutually exclusive assignment across interaction types.

## 4 Experiments

In this section, we outline the experimental setup used to evaluate LINKER, including data set preparation, baseline comparisons, and performance on the held-out test sets. Additional implementation details are provided in Supporting Information G, while the evaluation metrics are described in Supporting Information H.

### 4.1 Data Sets

We evaluated LINKER on protein–ligand complexes curated from PDBBind^21^ and BindingDB,^22^ both of which provide three-dimensional structures encompassing diverse binding interactions. Rather than using these data sets solely for binding affinity prediction, we derived residue-functional group interaction labels by decomposing ligands into functional groups and extracting noncovalent contacts using PLIP.^2^

#### 4.1.1 Atom-to-Functional-Group Label Construction

PLIP annotates noncovalent protein–ligand interactions at the atomic level from experimentally resolved complex structures. Let $A=\left\{a_{1},...,a_{N}\right\}$ denote the set of ligand atoms and $T=\left\{t_{1},...,t_{7}\right\}$ the set of interaction types considered in this work. Each atom *a*_*i*_ is encoded by a multihot interaction vector $y_{a_{i}}\in {\left\{0,1\right\}}^{\left|T\right|}$ with

17 $${\left(y_{a_{i}}\right)}_{k}=\{\begin{array}{ll} 1, & \mathrm{if}\;\mathrm{atom}\;a_{i}\;\mathrm{participates}\,\;\mathrm{in}\,\;\mathrm{interaction}\,\;t_{k},\\ 0, & \mathrm{otherwise} \end{array}$$

Independently, FGParser decomposes each ligand into a set of functional groups $G=\left\{g_{1},...,g_{M}\right\}$ and defines a many-to-many mapping $\phi :A\to 2^{G}$ , as atoms may belong to multiple functional groups. For a functional group *g*_*j*_, we define the associated atom set

18 $$A\left(g_{j}\right)=\left\{a_{i}\in A|g_{j}\in \phi \left(a_{i}\right)\right\}$$

To align atomic-level annotations with functional-group-level supervision, the interaction label of group *g*_*j*_ is computed by aggregating the atomic labels via an element-wise logical OR operation

19 $$y_{g_{j}}=\underset{a_{i}\in A\left(g_{j}\right)}{\vee }y_{a_{i}}$$

or equivalently, for each interaction type *t*_*k*_

20 $${\left(y_{g_{j}}\right)}_{k}=\max_{a_{i}\in A\left(g_{j}\right)}{\left(y_{a_{i}}\right)}_{k}$$

Functional groups without associated PLIP interactions are assigned the zero vector, **y**_*g*_*j*__ = **0**. This formulation preserves all atomic-level interaction evidence while enabling LINKER to operate at a chemically meaningful functional-group resolution.

Given their distinct characteristics, we adopted data set-specific splitting strategies. For PDBBind, which contains numerous experimentally resolved protein–ligand complexes, we applied LP-PDBBind,^23^ which is constructed from PDBbind v2020 and clusters complexes based on structural and sequence similarity to eliminate redundancies between training and test sets. This strategy is particularly critical for LINKER, whose supervision relies on three-dimensional structural motifs; preventing overlap ensures that the model captures generalizable sequence-to-interaction relationships rather than memorizing data set-specific patterns.

In contrast, BindingDB-derived docked complexes are characterized by fewer available samples, each target being associated with multiple ligands, and include synthetic structures generated through docking procedures, which may introduce data set-specific biases. To mitigate information leakage while preserving sufficient training data, we adopted a data set-specific splitting strategy tailored to these characteristics. Specifically, protein–ligand complexes were assigned to training, validation, and test sets according to the protocol outlined in Supporting Information B. This procedure prevents overlap at both the protein and ligand levels between splits, enabling an unbiased evaluation of model generalization in docking-based scenarios.

### 4.2 Baselines

In most prior work, protein–ligand interaction maps are generated only indirectly, as a secondary byproduct of models primarily optimized for drug-target affinity (DTA) or drug-target interaction (DTI) prediction. These models often rely on post hoc interpretability techniques such as attention visualization or gradient-based saliency, rather than being explicitly trained to predict interactions. To the best of our knowledge, LINKER is the first sequence-based model explicitly trained to predict biologically defined protein–ligand interactions, specifically fine-grained residue-functional group associations, rather than using interaction prediction as a secondary analysis.

To ensure a fair comparison, we benchmark against several well-established, state-of-the-art sequence-based models: ArkDTA^8^ and SPRINT,^5^ both of which are designed for DTA prediction, and HoTS,^10^ which is trained for DTI prediction. Although these baselines do not focus on interaction prediction as a primary objective, they produce interpretable interaction maps as auxiliary outputs, making them relevant for comparison in terms of input modality and interpretability scope. By evaluating against these models, our objective is to highlight the benefits of LINKER’s targeted supervision and its ability to learn chemically meaningful and interpretable interaction patterns from sequence alone.

### 4.3 Results

#### 4.3.1 Residue Interaction Prediction

We evaluated the predictive performance of LINKER in identifying interaction residues, i.e., protein residues involved in ligand binding, using hard binary labels derived from PLIP annotations. To enable a fair comparison with baseline models that are supervised only at the residue level, we aggregated our finer-grained residue-functional group interaction labels into residue-level binary indicators, where a residue is marked as positive if it interacts with at least one ligand atom. We then compared LINKER against baselines using precision-recall (PR) and receiver operating characteristic (ROC) curves computed over all protein residues. These metrics capture model performance under class imbalance and evaluate overall discriminative power in distinguishing interacting from noninteracting residues.

To enable this comparison, we derive residue-level interaction scores from LINKER’s output tensor defined in Section 2, where the model predicts a tensor **P** ∈ [0, 1]^*R*×*F*×7^ representing interaction probabilities between *R* protein residues and *F* ligand functional groups in seven biologically defined interaction types. We first aggregate over functional groups by taking the maximum along the *F*-dimension

21 $$U_{r,k}=\max_{1\leq f\leq F}P_{r,f,k},,\mathrm{for}\,\;r=1,...,R\,\;\mathrm{and}\,\;k=1,...,7$$

resulting in a residue-wise interaction score matrix **U** ∈ [0, 1]^*R*×7^. Next, we aggregate over interaction types

22 $$y_{r}=\max_{1\leq k\leq 7}U_{r,k},,\mathrm{for}\,\;r=1,...,R$$

which produces a final prediction vector at the residue level **y** ∈ [0, 1]^*R*^. This transformation produces a single interaction confidence score per residue, enabling a fair comparison with the baselines, which is supervised at the residue level. Importantly, this reduction preserves the fine-grained interaction information learned by LINKER while aligning it with the coarser evaluation setting.

As shown in Figure 2, LINKER consistently outperforms all baseline models across both data sets in terms of both PR and ROC curves. On the PDBBind data set, LINKER achieves an average precision (AP) of 0.4076, significantly surpassing ArkDTA (0.2941), HoTS (0.0685), and SPRINT (0.0335), despite the extreme class imbalance (positive prevalence, prev = 2.4%). The ROC AUC scores follow a similar trend, with LINKER reaching 0.9369, ahead of ArkDTA (0.8689), HoTS (0.6667), and SPRINT (0.6024), indicating strong discriminative power. Performance remains robust on the BindingDB benchmark, where LINKER again leads with an AP of 0.2976 and an AUC of 0.9017. In comparison, ArkDTA achieves 0.2496 (AP) and 0.8155 (AUC), HoTS achieves 0.0430 (AP) and 0.5793 (AUC), and SPRINT achieves 0.0388 (AP) and 0.6084 (AUC). These results confirm the effectiveness of LINKER’s interaction supervision strategy, showing that it can extract biologically meaningful signals from sequence inputs alone and generalize well to unseen protein–ligand pairs.

**Figure 2:**
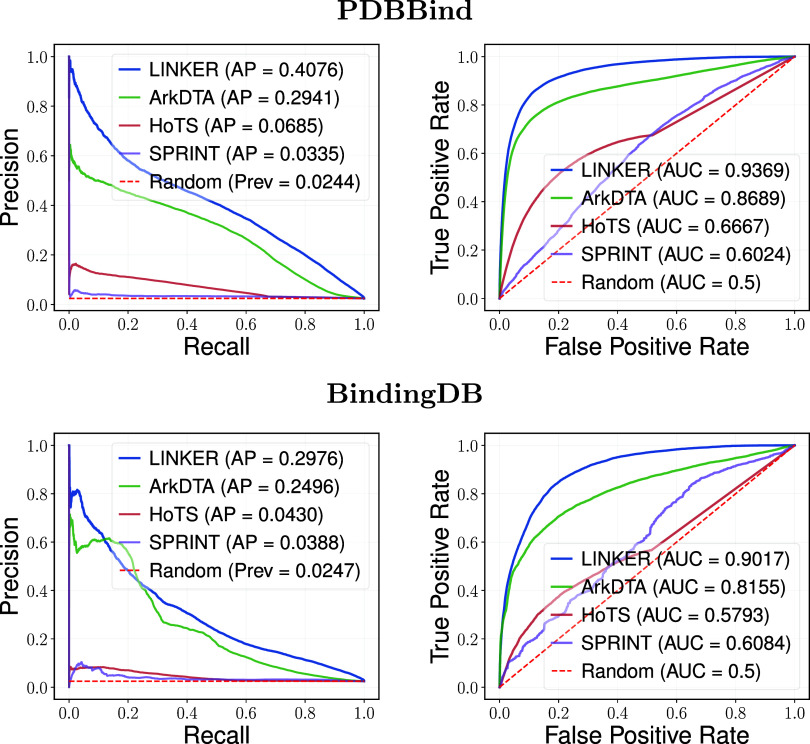
LINKER accurately identifies protein–ligand interaction residues directly from sequences. Evaluation using PR and ROC curves at the residue level shows that LINKER consistently outperforms baseline models and random predictions across both data sets, demonstrating robust performance under class imbalance and strong overall discriminative ability.

#### 4.3.2 Residue Interaction Prediction with Soft Labels

While PLIP-derived interaction labels offer reliable supervision, they are based on static snapshots of crystallized protein–ligand complexes and rely on rigid geometric cutoffs. This binary labeling scheme fails to capture the dynamic nature of molecular recognition, where interactions may transiently form and break due to conformational flexibility, induced fit, or thermal fluctuations. In reality, ligand binding often involves clusters of spatially and sequentially adjacent residues that collectively contribute to binding affinity, even if only a subset is captured at a single time point. To better reflect biological continuity and mitigate artifacts introduced by hard distance thresholds, we applied Gaussian kernel smoothing along the residue dimension, softening binary labels into a more realistic distribution of interaction probabilities. Concretely, let us denote

23 $$y_{\mathrm{hard}}\left[i\right]=I\left[\sum_{j}Y_{i,j}>0\right]$$

as the binary residue-level label from PLIP. The smoothed label for the residue *i* is then defined as

24 $$y_{\mathrm{smooth}}\left[i\right]=\{\begin{array}{ll} \max_{c\in H}\exp\left(-\frac{{\left(i-c\right)}^{2}}{2\sigma^{2}}\right), & H\neq Ø,\\ 0, & H=Ø \end{array}$$

where *H* = {*c* | *y*_hard_[*c*] = 1}.

Here, PLIP-identified residues serve as anchor points with full confidence, while their immediate neighbors receive decaying support smoothly. This smoothing does not alter the underlying ground truth labels; rather, it acts as a biologically motivated regularization that reflects the clustered nature of binding sites. By mitigating cutoff-induced artifacts, it produces graded signals that enable a more flexible and faithful evaluation of interpretability. A visualization of this smoothing procedure, based on the above formulas, can be found in Supporting Information C.

This soft labeling approach reflects the reality that interaction signals are often spatially and sequentially diffuse, rather than confined to discrete residue positions. To assess how well each model captures this broader interaction context, we computed weighted precision of residue-functional group predictions at varying confidence thresholds across multiple smoothing levels. As shown in Figure 3, under minimal smoothing (Smoothing 1), which closely approximates hard PLIP-derived binary supervision, performance across all models remains relatively low and varies weakly with confidence threshold, reflecting the sparsity and rigidity of hard binary supervision. As the smoothing scale increases, with strong smoothing (Smoothing 4) corresponding to a substantially broadened residue interaction neighborhood, weighted precision improves for all methods, indicating that soft residue interaction labels provide a more informative and biologically consistent evaluation signal. Notably, LINKER exhibits pronounced sensitivity to the confidence threshold, with precision peaking at intermediate thresholds and decreasing at stricter cutoffs, suggesting that its predicted confidence scores are finely ranked and responsive to selective thresholding. In contrast, ArkDTA and other baselines show comparatively flatter trends across thresholds, implying weaker score stratification and limited responsiveness to changes in confidence criteria. These observations suggest that Gaussian smoothing not only enhances overall performance but also reveals differences in how models assign and calibrate confidence to residue-functional group interactions.

**Figure 3:**
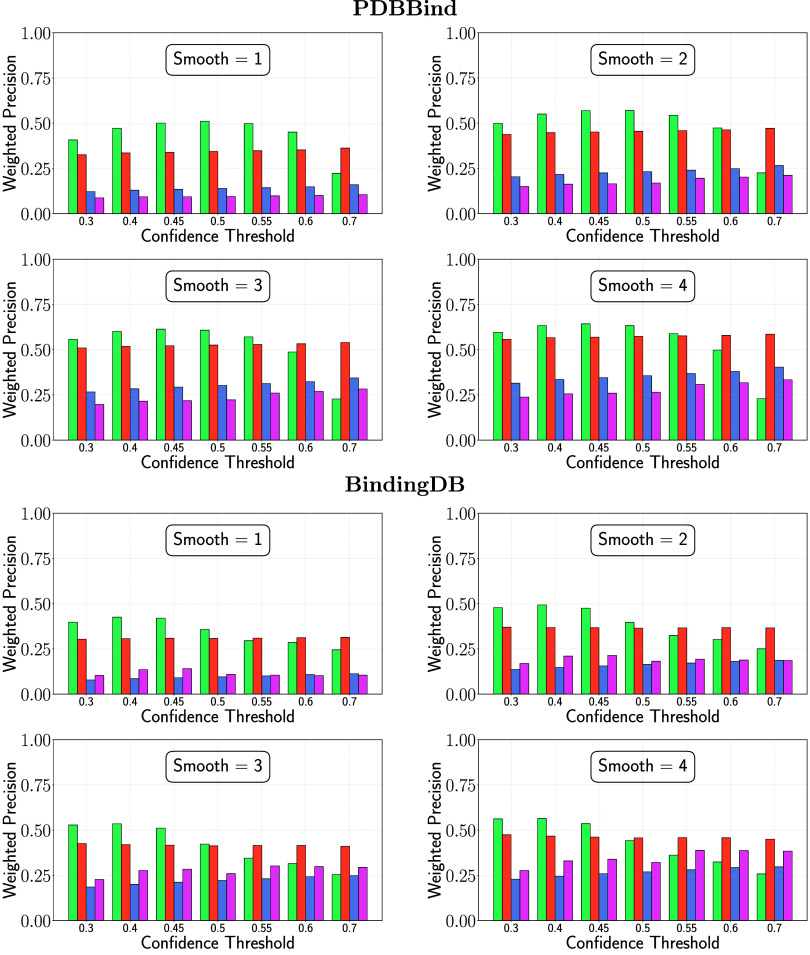
Performance under soft residue interaction supervision across smoothing scales. Weighted precision of residue-functional group predictions is evaluated across multiple confidence thresholds under increasing Gaussian smoothing levels. Results are shown for LINKER (teal), ArkDTA (coral), HoTS (cornflower blue), and SPRINT (pink).

#### 4.3.3 Residue-Functional Group Interaction Prediction

We evaluated LINKER’s ability to predict fine-grained interactions between protein residues and ligand functional groups, a novel task that, to our knowledge, has not been directly addressed in prior work. To evaluate LINKER’s performance on this new task, we compare its predictions against ground truth labels derived from PLIP and report both quantitative metrics and qualitative visualizations.

##### 4.3.3.1 Quantitative Evaluation

Figure 4 presents LINKER’s performance on residue-functional group interaction prediction across both the PDBBind and BindingDB data sets. In each case, the precision-recall (PR) curves are shown alongside random baselines, with prevalences of 0.000613 and 0.000596, respectively, reflecting the extreme sparsity of positive labels. To account for this class imbalance, model performance is summarized using average precision (AP) and fold enrichment, defined as the ratio of precision to prevalence. These metrics are computed on a linear scale, although precision is plotted logarithmically to better visualize low values under this highly imbalanced setting.

**Figure 4:**
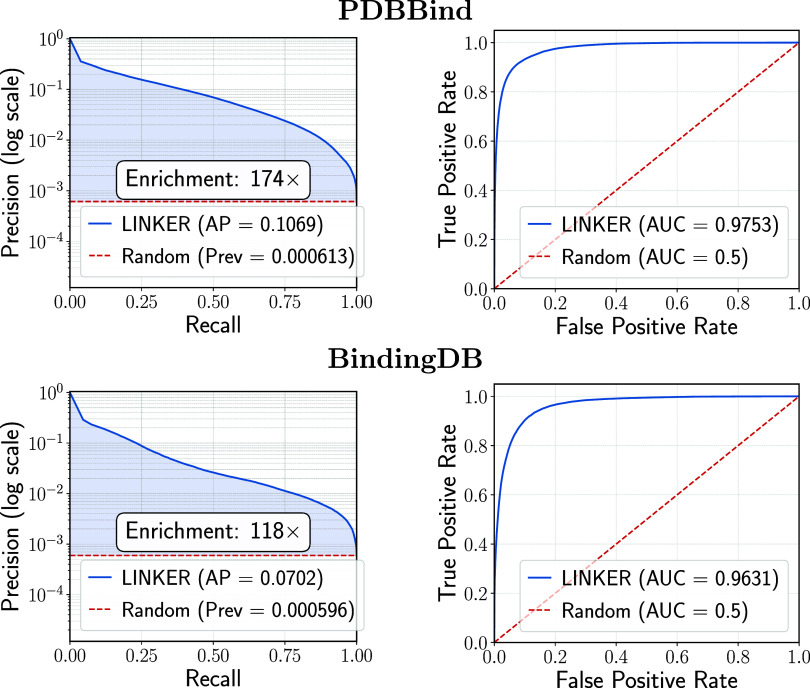
LINKER predicts fine-grained residue-functional group interactions with high precision and recall despite extreme data sparsity. Evaluation of LINKER’s ability to identify specific biochemical interactions between protein residues and ligand functional groups across two data sets. The PR curves show marked enrichment over the random baseline, while the ROC curves confirm strong overall discrimination.

On the PDBBind benchmark, LINKER achieves an AP of 0.1069 and up to 174× enrichment over random, demonstrating its effectiveness in recovering true interactions despite the rarity of positives. On BindingDB, LINKER maintains strong performance with an AP of 0.0702 and 118× enrichment, further confirming the model’s ability to generalize across data sets with distinct chemical diversity. The corresponding ROC curves indicate excellent discrimination capabilities, with AUC scores of 0.9753 on PDBBind and 0.9631 on BindingDB. Together, these results confirm LINKER’s robustness in detecting rare, fine-grained biochemical interactions from sequence data alone and underscore its generalizability across structurally diverse benchmarks.

##### 4.3.3.2 Qualitative Evaluation

To qualitatively assess the interpretability and robustness of LINKER’s predictions across diverse structural contexts, we selected a small set of representative protein–ligand complexes chosen to highlight the model’s ability to recover chemically and structurally meaningful interaction patterns. All qualitative results shown here are generated using LINKER trained on the PDBBind data set. These examples span common and challenging molecular recognition scenarios, including a protein with a relatively simple architecture (PDB ID: 5eis,), a larger and more structurally complex protein (PDB ID: 3e63), and a single protein interacting with chemically distinct ligands (PDB IDs: 4q83 and 5n24), enabling systematic examination of model behavior across variations in protein complexity and ligand chemistry. For completeness, we additionally report and analyze representative challenging prediction cases in Supporting Information I.2, highlighting scenarios in which the model does not fully recover correct interaction patterns and discussing potential underlying causes.

Figure 5 presents predicted residue–functional group interaction maps for these complexes, compared directly with PLIP-derived ground-truth annotations. LINKER consistently highlights spatially localized interaction regions that align well with annotated contacts, assigning the highest confidence to residue–group pairs in close spatial proximity to the true interactions. This demonstrates the model’s ability to infer chemically meaningful residue–group associations from sequence-level input while maintaining consistency with structural plausibility. Notably, even in complexes with sparse ground-truth labels, LINKER suppresses spurious predictions, indicating robust generalization.

**Figure 5:**
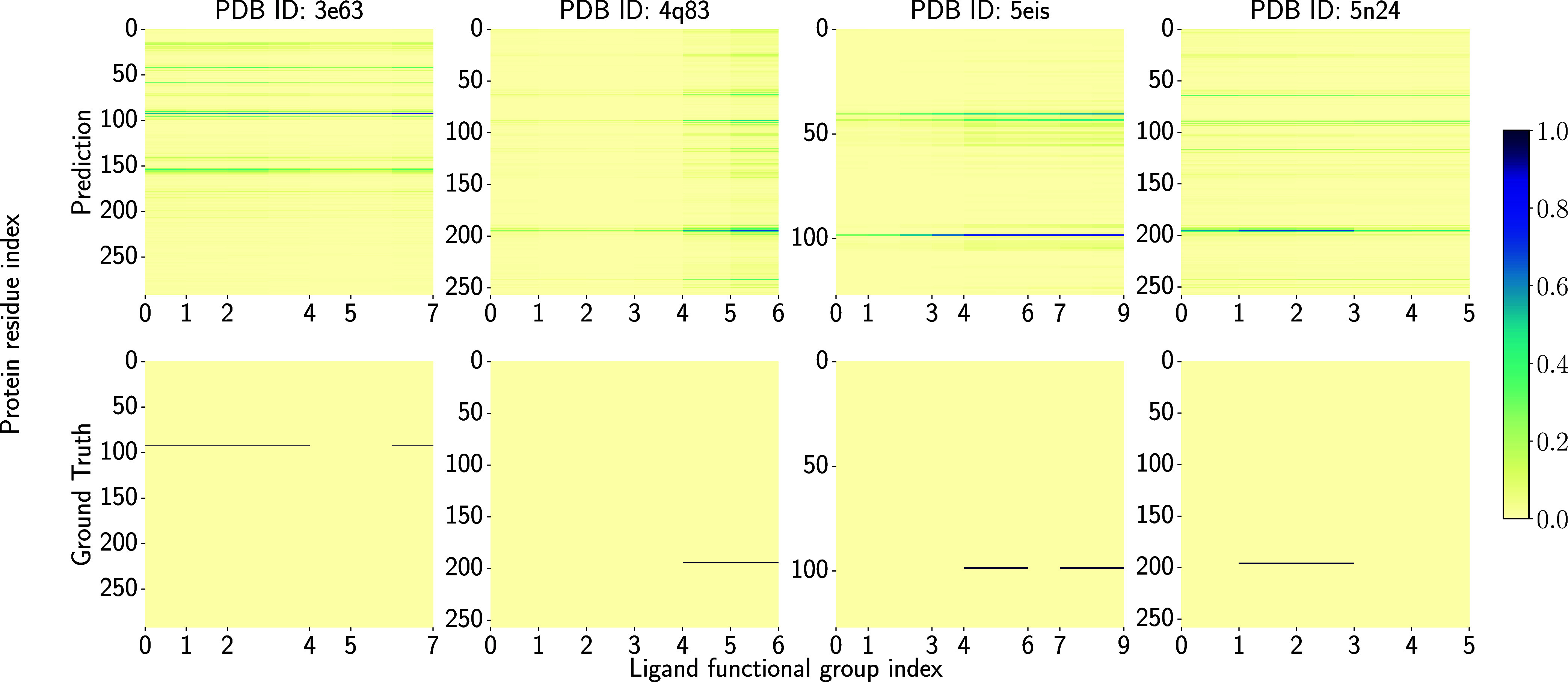
Predicted interaction maps by LINKER align with ground-truth structural annotations. Qualitative comparison between LINKER-predicted residue–functional group interaction maps and PLIP-derived ground-truth annotations for hydrogen bonds across four protein–ligand complexes. The heatmaps depict interaction probabilities between protein residue indices (*y*-axis) and ligand functional group indices (*x*-axis). LINKER consistently identifies spatially localized interaction regions that correspond closely to the annotated contacts, demonstrating its ability to generate chemically interpretable and structurally plausible interaction patterns from sequence-level input alone.

Figure 6 provides complementary three-dimensional visualizations for proteins with simple and complex architectures (PDB IDs: 5eis and 3e63). These views illustrate how predicted binding probability profiles along the protein sequence correspond to structurally coherent binding pockets in both compact and highly structured proteins, highlighting LINKER’s robustness to differences in protein size and architectural complexity. Figure 7 examines ligand-dependent binding behavior by visualizing the same protein interacting with chemically distinct ligands (PDB IDs: 4q83 and 5n24). The results show that LINKER adapts its predicted interaction patterns in response to ligand chemistry, capturing shifts in binding regions that are consistent with the underlying protein–ligand complexes. Together, these complementary two-dimensional interaction maps and three-dimensional structural visualizations demonstrate that LINKER produces interpretable and biologically grounded predictions across diverse molecular recognition scenarios.

**Figure 6:**
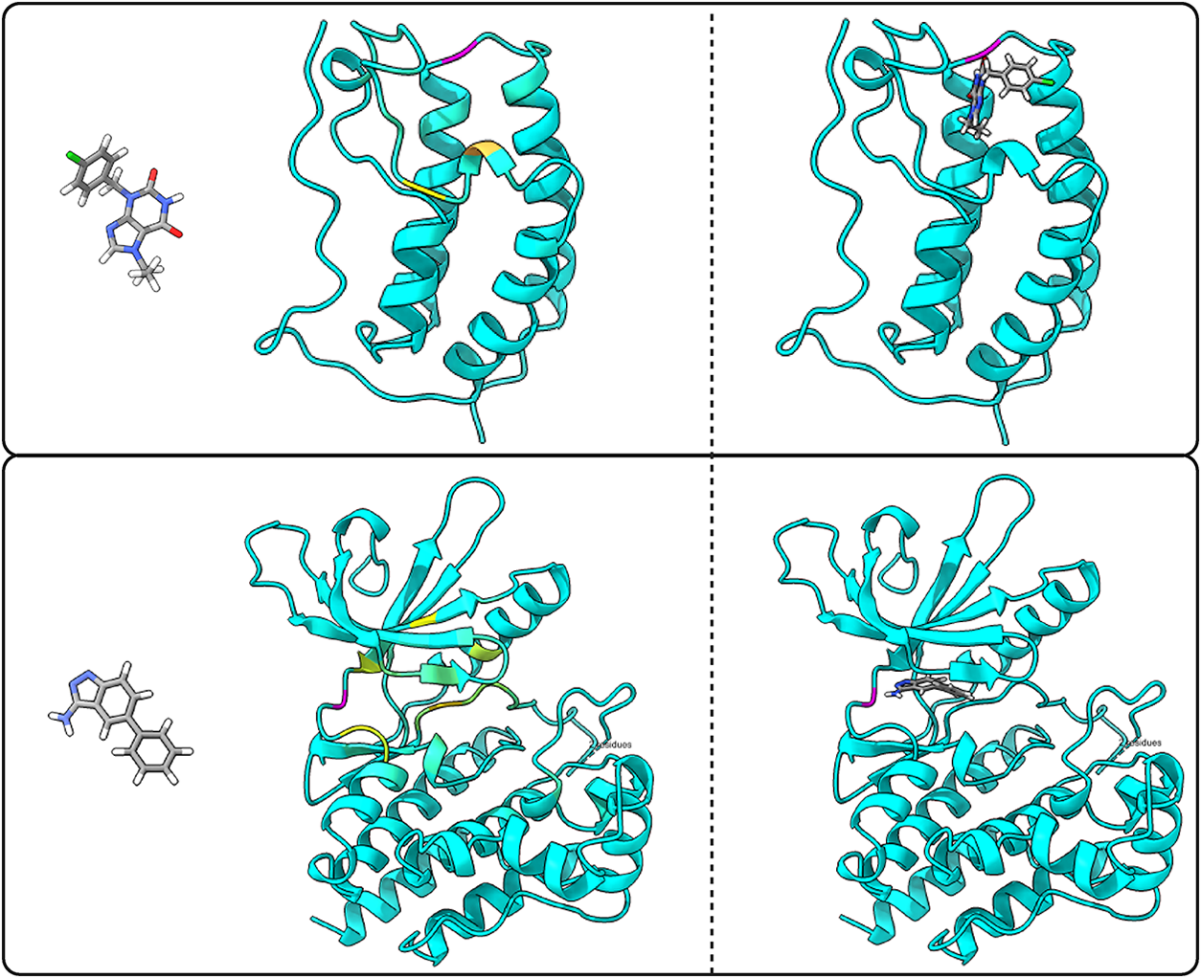
Qualitative case studies highlighting the effect of protein structural complexity. Representative complexes spanning a protein with a relatively simple architecture (PDB ID: 5eis, top) and a larger, more structurally complex protein (PDB ID: 3e63, bottom). From left to right, the ligand structure, the predicted binding probability aggregated across all ligand functional groups along the protein sequence (values range from 0 to 1 and are color-coded from cyan to yellow to magenta), and the corresponding protein–ligand complex are shown. These examples illustrate LINKER’s ability to localize binding regions across varying levels of protein structural complexity.

**Figure 7:**
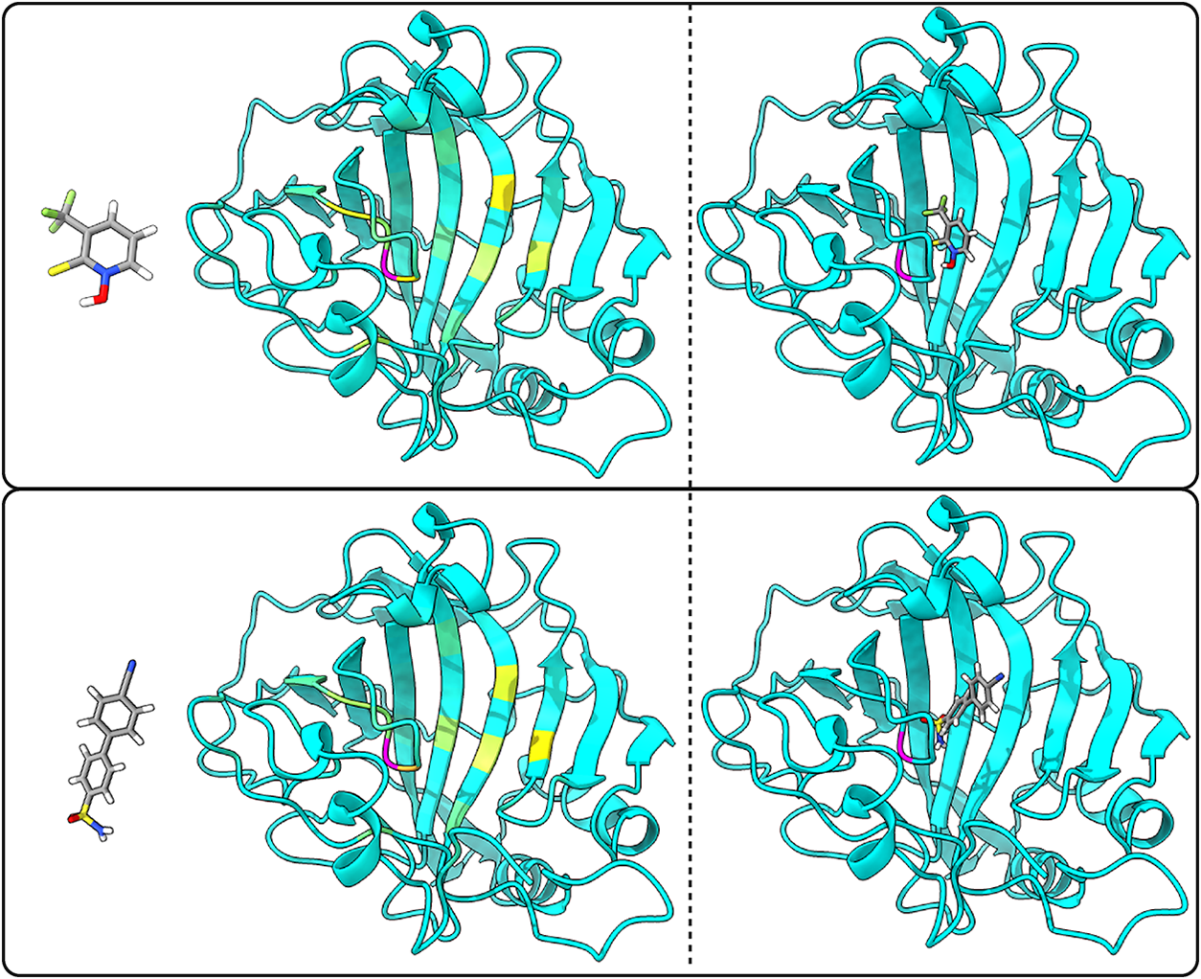
Qualitative case studies highlighting ligand-dependent interaction patterns for the same protein. Representative complexes of the same protein interacting with two chemically distinct ligands (PDB IDs: 4q83, top, and 5n24, bottom). From left to right, the ligand structure, the predicted binding probability aggregated across all ligand functional groups along the protein sequence (values range from 0 to 1 and are color-coded from cyan to yellow to magenta), and the corresponding protein–ligand complex are shown. These examples demonstrate LINKER’s capacity to adapt predicted interaction patterns in response to changes in ligand chemistry.

### 4.4 Comparison with Conventional Structure-Docking-Interaction Pipelines

To contextualize the practical utility of LINKER, we compare it with a conventional structure-based workflow commonly used for protein–ligand interaction analysis in classical drug discovery. This pipeline consists of three sequential stages: protein structure prediction from amino acid sequence using AlphaFold2,^24^ ligand docking using AutoDock Vina,^1^ and atom-level interaction analysis of the resulting complexes using PLIP.^2^ Due to the substantial computational cost and runtime associated with this multistage pipeline, we restrict this comparison to the same four representative protein–ligand pairs used in the qualitative analysis described in Section 4.3.3. Both LINKER and the AlphaFold2–Vina–PLIP pipeline were evaluated on these cases, with identical protein sequences and ligand SMILES provided as inputs.

Figure 8 summarizes the outputs of the structure-based pipeline and enables a direct qualitative comparison with LINKER. The top row shows alignments between AlphaFold2-predicted structures and experimentally resolved protein structures, with low Root Mean Square Deviation (RMSD) values across all cases, indicating that the predicted models provide reasonable structural scaffolds for docking. In the conventional pipeline, ligands were docked into the predicted structures using AutoDock Vina, and interaction patterns were extracted using PLIP for the top ten ranked docking poses. These interaction patterns are visualized using color to indicate docking pose rank as a proxy for confidence, reflecting the variability introduced by pose enumeration and ranking.

**Figure 8:**
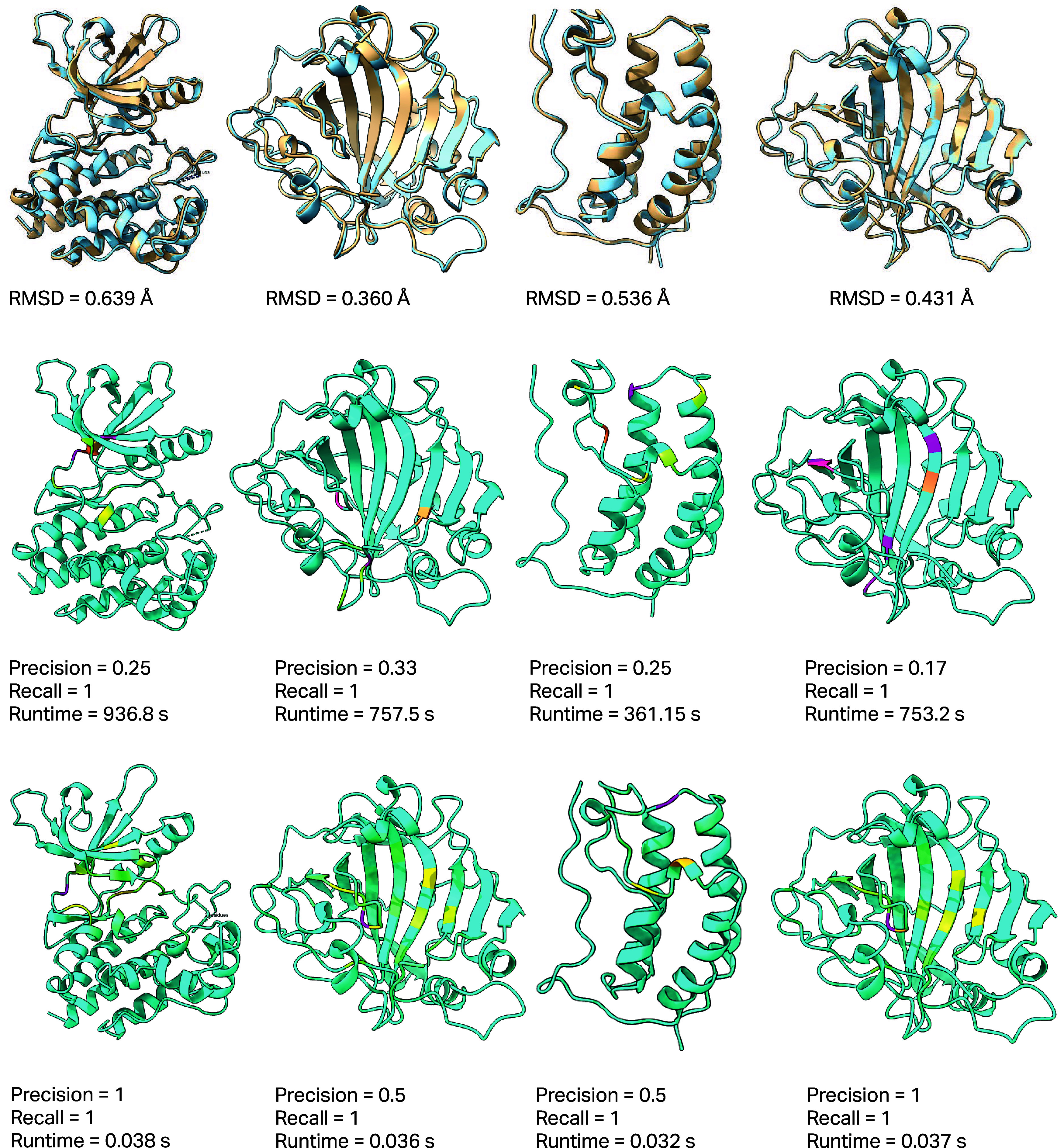
Comparison between a conventional structure–docking pipeline and LINKER. Top row: Alignment of AlphaFold2-predicted protein structures (tan) with experimentally resolved structures (cyan), with RMSD values shown for each case. Middle row: Interaction patterns derived from PLIP analysis of the top ten AutoDock Vina poses docked into the predicted structures, where color encodes docking pose rank. Bottom row: Interaction maps produced by LINKER for the same protein–ligand pairs, where color encodes predicted binding probability. For both rows, colors span a normalized range from 0 to 1 and are shown from cyan to yellow to magenta, but represent different underlying quantities.

For the same protein–ligand pairs, LINKER directly generates interaction maps from protein sequence and ligand SMILES alone, without relying on explicit structure prediction or docking. Despite the fundamentally different inference processes, both approaches consistently achieve a recall of 1 across all evaluated cases when using a probability threshold of 0.5, indicating that all ground-truth interacting residues are successfully recovered. This suggests that both the conventional structure-based pipeline and LINKER are highly sensitive to true interaction regions. However, notable differences emerge in precision and computational cost. As shown in Figure 8, the structure–docking pipeline exhibits relatively low precision, ranging from 0.17 to 0.33, reflecting the presence of spurious interaction signals arising from docking pose variability and uncertainty. In contrast, LINKER achieves substantially higher precision, ranging from 0.5 to 1.0, producing more selective and spatially concentrated interaction maps. These accuracy differences are accompanied by a dramatic disparity in runtime: the structure-based pipeline requires hundreds of seconds per complex due to the cumulative cost of structure prediction, docking, and PLIP analysis, whereas LINKER produces interaction maps within a few hundredths of a second. Together, these results indicate that while both approaches reliably recover true interaction regions, LINKER provides more precise interaction localization with orders-of-magnitude improvements in computational efficiency.

### 4.5 Evaluation of LINKER Representations

To assess the generality and biological relevance of the representations learned by LINKER, we evaluated their effectiveness in a downstream binding affinity prediction task. This experiment tests whether interaction-aware features, centered on residue-functional group associations, encode transferable biochemical information beyond their original supervision objective. Although LINKER is not trained explicitly for affinity prediction, we use its learned representations as input to a separate predictor and benchmark performance against sequence-based baselines under identical experimental conditions. In addition, we include traditional structure-based and classical machine learning methods, as well as multimodal approaches at the protein-level that leverage additional structural information to contextualize LINKER’s performance. These comparisons provide a broader context for interpreting LINKER’s results alongside methods that rely on different data modalities and training objectives. The architecture and training details of the *Binding Affinity Predictor* are provided in Supporting Information E.

Tables 1 and Table 2 report binding affinity prediction performance on PDBBind and BindingDB using Root Mean Square Error (RMSE), Pearson correlation coefficient (*R*), and Concordance Index (CI). RMSE measures absolute regression accuracy and calibration, whereas *R* and CI evaluate linear association and relative affinity ranking, which is especially important for virtual screening. The results show that calibration and ranking performance can differ substantially across data sets.

**Table 1:** Binding Affinity Prediction Performance on the PDBBind Test Sets^a^

model	RMSE	*R*	CI
AutoDock Vina^1^	2.56 (0.00)	0.4542 (0.00)	0.6217 (0.00)
InteractionGraphNet (IGN)^25^	2.16 (0.13)	0.5231 (0.086)	0.6454 (0.030)
Random Forest (RF)-Score^26^	2.10 (0.003)	0.5822 (0.000035)	0.7085 (0.000020)
DeepDTA^27^	2.29 (0.04)	0.4920 (0.053)	0.6582 (0.0058)
MPRL^28^	1.55 (0.08)	0.5332 (0.030)	0.6745 (0.0015)
StructureFree-DTA^29^	1.49 (0.004)	0.5450 (0.0018)	0.6810 (0.000094)
DualPG-DTA^30^	1.51 (0.05)	0.5284 (0.023)	0.6732 (0.0010)
SSM-DTA^31^	1.62 (0.06)	0.5056 (0.027)	0.6678 (0.0012)
SPRINT^5^	1.50 (0.02)	0.5173 (0.00060)	0.6697 (0.000034)
HoTS^10^	1.67 (0.04)	0.4328 (0.0012)	0.6348 (0.000068)
ArkDTA^8^	1.48 (0.07)	0.5398 (0.0021)	0.6784 (0.00012)
LINKER (ours)	1.48 (0.01)	0.5098 (0.00058)	0.6691 (0.000019)

a RMSE, *R* and CI are reported as mean (standard deviation) over three independent runs. Numbers in bold indicate the best performance in each column.

**Table 2:** Binding Affinity Prediction Performance on the BindingDB Test Sets^a^

model	RMSE	*R*	CI
InteractionGraphNet (IGN)^25^	2.81 (0.20)	0.5650 (0.11)	0.6221 (0.037)
Random Forest (RF)-Score^26^	3.31 (0.007)	0.4956 (0.000091)	0.6756 (0.00026)
DeepDTA^27^	3.66 (0.22)	0.4023 (0.16)	0.6287 (0.032)
MPRL^28^	3.71 (0.08)	0.3725 (0.035)	0.6128 (0.0045)
StructureFree-DTA^29^	3.54 (0.12)	0.4115 (0.057)	0.6392 (0.019)
DualPG-DTA^30^	3.58 (0.15)	0.4187 (0.068)	0.6415 (0.0083)
SSM-DTA^31^	3.63 (0.19)	0.3982 (0.082)	0.6299 (0.010)
SPRINT^5^	1.39 (0.05)	0.9277 (0.0066)	0.8868 (0.00083)
HoTS^10^	3.69 (0.06)	0.3524 (0.0078)	0.5989 (0.0010)
ArkDTA^8^	4.20 (0.08)	0.0767 (0.017)	0.5106 (0.0014)
LINKER (ours)	3.00 (0.01)	0.5880 (0.0084)	0.6894 (0.0024)

a RMSE, *R* and CI are reported as mean (standard deviation) over three independent runs. Numbers in bold indicate the best performance in each column.

On PDBBind, RF-Score achieves the highest *R* and CI but has a larger RMSE, suggesting strong preservation of relative affinity trends but weaker absolute calibration. In contrast, modern structure-free DTA models such as StructureFree-DTA and ArkDTA obtain lower RMSE values, reflecting better quantitative calibration. LINKER also achieves one of the lowest RMSE values on PDBBind while maintaining moderate *R* and CI. Since LINKER is trained to predict residue-functional group interactions rather than binding affinity directly, these results indicate that its interaction-aware representations capture biochemical signals transferable to affinity prediction.

On BindingDB, SPRINT performs best across all three metrics, likely due to its direct optimization for affinity regression and better alignment with the benchmark label distribution. BindingDB in this study uses docked protein–ligand complexes rather than experimentally resolved structures, making its distribution different from PDBBind. IGN obtains relatively low RMSE by using explicit complex geometry, but its lower *R* and CI compared with SPRINT and LINKER suggest that 3D structural information alone is insufficient without suitable training objectives and data set alignment.

Several baselines show clear cross-data set degradation. For example, ArkDTA performs well on PDBBind but drops substantially on BindingDB, with near-random CI, and HoTS shows a similar trend. In contrast, LINKER maintains more stable *R* and CI across both data sets. Although its BindingDB RMSE remains higher than affinity-specialized models, its preserved CI suggests meaningful relative affinity ordering. Overall, these results support the transferability of LINKER’s residue-functional group representations for downstream affinity-related ranking tasks.

Figure 9 complements Tables 1 and 2 by visually assessing dispersion and monotonic trends between predicted and reference binding affinities for representative interpretable models. Across both data sets, the scatter plots reveal differences in calibration and ranking behavior that are not fully captured by scalar metrics alone. LINKER exhibits moderate but consistent positive trends on both PDBBind and BindingDB, supporting the observation that its interaction-aware representations preserve affinity-related ordering to a nonrandom extent when transferred to downstream evaluation.

**Figure 9:**
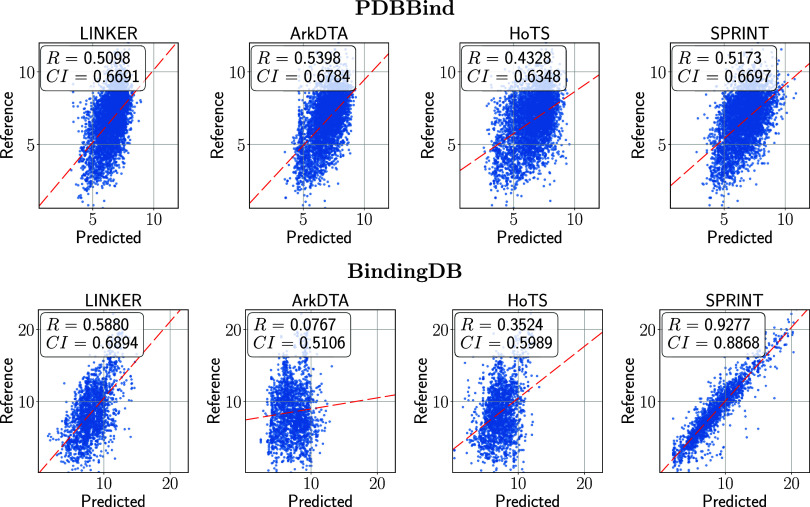
Transferability of interpretable affinity-related representations across PDBBind and BindingDB. Each point represents a test sample with predicted and reference binding affinities. The dashed red line denotes the linear regression fitted between predictions and reference values. Predictions are averaged over three independent runs with different random seeds. *R* and CI are reported to assess linear association and ranking performance, respectively.

To further assess the quality and interpretability of LINKER’s learned chemical representations, we visualized ligand functional group embeddings generated by the FINGER-ID encoder using t-SNE. Figure 10 illustrate the robustness and chemical coherence of these embeddings across different data sets and data splits. Functional groups belonging to broader chemical families (e.g., alcohols, ethers, amines, and amides) exhibit a consistent and hierarchical spatial organization in the latent space. The reproducible positioning of specific functional group centroids within each family across all splits and data sets indicates that LINKER captures intrinsic, chemically meaningful relationships rather than overfitting to data set or split-specific patterns. The complete t-SNE visualization of all functional groups is provided in Supporting Information I.1.

**Figure 10:**
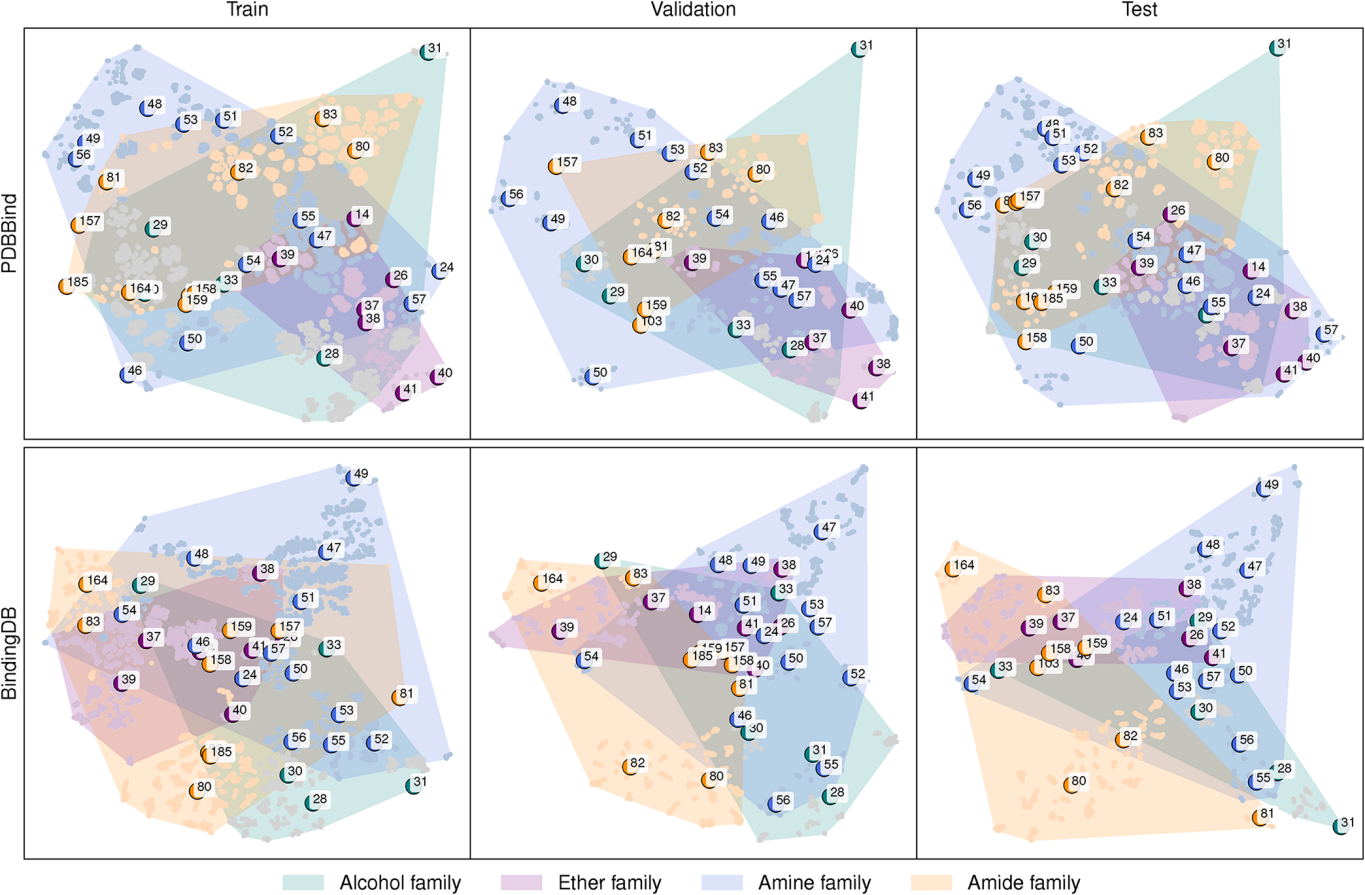
Stability and hierarchical organization of functional group embeddings within chemical families. t-SNE projects illustrate the latent space distribution of ligand functional groups encoded by FINGER-ID, arranged by data set and data partition. Shaded polygonal regions indicate four major chemical families: alcohols, ethers, amines, and amides. Individual points represent specific functional group instances, and numbered circles indicate centroids of distinct functional group types labeled with their PyCheckMol identifiers. The spatial arrangement of these specific groups is shown to be reproducible within their broader chemical families across all splits and data sets, and confirms that our model captures intrinsic, chemically grounded hierarchical relationships.

## 5 Conclusion

We introduced LINKER, a sequence-based framework for predicting biologically defined interaction types between protein residues and ligand functional groups. By leveraging structure-supervised attention trained on functional group abstractions, LINKER eliminates the need for 3D complex structures at inference time, a key limitation of existing structure-dependent methods, while directly modeling chemically meaningful interactions such as hydrogen bonding and π-stacking from sequence alone. In contrast to approaches that attempt to explain binding affinity predictions post hoc, LINKER adopts a fundamentally different paradigm. It explicitly predicts interaction patterns as the primary modeling objective, the mechanistic basis of molecular recognition, and subsequently leverages these interpretable signals to support downstream tasks such as affinity estimation. This shift from global regression to local interaction modeling more closely reflects the underlying biophysics of protein–ligand binding. Extensive evaluation on the LP-PDBBind and BindingDB benchmarks shows that LINKER produces accurate, fine-grained interaction maps that align with known biochemical principles, even in highly data-sparse regimes. Moreover, the learned representations show evidence of transferability, preserving affinity-related ranking signals in downstream evaluation despite not being optimized for binding affinity regression.

At the same time, the current framework represents an intentionally focused first step toward a broader interaction-centric modeling paradigm, leaving several important directions for future work. Beyond extending LINKER to explicitly model metal-mediated interactions and incorporating chemically accurate, context-dependent ligand states such as protonation, charge, and stereochemistry, an important next step is the systematic interpretation of additional protein regions that receive elevated interaction scores beyond the annotated primary binding site. Characterizing whether these regions correspond to secondary or allosteric pockets, transient binding environments, or false positives remains challenging due to limited ground truth annotations, but offers opportunities to refine interaction prediction and enable the discovery of novel binding sites. In addition, integrating LINKER as a component within broader drug discovery pipelines, such as virtual screening and lead optimization workflows, represents a promising direction, where predicted interaction maps could guide binding site identification, SAR interpretation, and functional group prioritization. Finally, since LINKER is centered on localized and interpretable interaction patterns, the framework is naturally extensible to other molecular recognition problems, including protein–protein, protein-RNA, and antibody–antigen interactions, underscoring its potential as a general foundation for interpretable and structure-free modeling of biomolecular interactions.

## Supplementary Material



## Data Availability

All data sets and source code used in this study are publicly available in our GitHub repository at https://github.com/HySonLab/LINKER.

## Notes

Functional group detection with PyCheckMol; data set construction and preparation; smooth label visualization; ligand representation ablation study; binding affinity predictor architecture and training procedures; implementation details and evaluation metrics; detailed t-SNE analysis of functional group embeddings; and qualitative examination of challenging prediction cases (PDF)
