# Supplementary material for: LINKER: Learning Interactions between Functional Groups and Residues with Chemical Knowledge‑Enhanced Reasoning and Explainability
Source: J Chem Inf Model. 2026 Jul 16;66(14):8579–93. doi: 10.1021/acs.jcim.6c00527 (PMC13417889; doi:10.1021/acs.jcim.6c00527)
Supplement: Supplementary file 1 [file ci6c00527_si_001.pdf]

LINKER: Learning Interactions Between Functional Groups and Residues With Chemical Knowledge-Enhanced Reasoning and Explainability (Supporting Information)

Phuc Pham ,[†] Viet Thanh Duy Nguyen ,[†] Kevin Song ,[‡] Jake Chen ,[¶] and Truong-Son Hy [*,†]

†*Department of Computer Science, The University of Alabama at Birmingham, Birmingham, AL 35294, United States*

‡*Department of Biomedical Engineering, The University of Alabama at Birmingham, Birmingham, AL 35294, United States*

¶*Department of Biomedical Informatics and Data Science, The University of Alabama at Birmingham, AL 35294, United States*

E-mail: thy@uab.edu

A Functional Group Detection with PyCheckMol

PyCheckMol is used in this work to identify functional groups from ligand structures based on a curated library of predefined chemical substructure patterns. These patterns encode functional group definitions at the atom and connectivity level and are matched against molecular graphs derived from SMILES representations.

Conceptually, PyCheckMol follows a rule-based substructure matching paradigm that is closely related to SMARTS-based pattern matching. However, the patterns are implemented internally using PyCheckMol's native representation and matching engine, rather than user-defined SMARTS expressions. This design enables consistent and reproducible functional group assignments without requiring manual pattern specification.

The current version of PyCheckMol employed in this study includes a library of 204 predefined functional group patterns, covering a broad range of chemical motifs commonly encountered in organic and medicinal chemistry. These groups span carbonyl compounds, alcohols, amines, carboxylic acid derivatives, heterocycles, sulfur- and phosphorus-containing groups, halogenated moieties, and selected organometallic motifs.

In terms of elemental coverage, the predefined patterns primarily focus on elements typically present in organic and medicinal chemistry compounds, including C, H, N, O, S, P, and halogens (F, Cl, Br, I). In addition, PyCheckMol provides limited support for specific metal-associated functional groups, which mainly correspond to organometallic motifs (e.g., organolithium and organomagnesium compounds).

B Dataset Construction and Preparation

We first collected samples consisting of protein sequences paired with their corresponding ligand SMILES representations from the BindingDB Docked Complexes (available at bindingdb.org), which include the corresponding 3D complexes of proteins and ligands pre-docked using Surflex.[1] The predocked BindingDB complexes provide an ideal and diverse source of data for extracting interaction labels for LINKER. While PDBBind reflects experimentally determined protein-ligand interactions, BindingDB complexes are generated computationally, providing an alternative perspective for evaluating LINKER. Due to the nature of the dataset, where a single protein is docked with multiple ligands and multiple proteins are included, special care is required to avoid data leakage and reduce bias. To address this, we employed

the pipeline illustrated in Figure 1 to split the data into training, validation, and test sets.

Protein Matching A protein matching module was applied to identify pairs of proteins with sequence similarity, which was computed via pairwise global alignments using the Needleman–Wunsch algorithm[2] with the BLOSUM62 substitution matrix.[3] Edges were then created between protein pairs exceeding a predefined similarity threshold τ to construct a protein similarity network. By grouping highly similar proteins, this module helps prevent overrepresentation of specific protein types within a split, thereby mitigating bias during model training. In our experiments, we set $\tau = 0.5$; gap opening and extension penalties were set to -10 and -0.5, respectively, and alignments were implemented using the BioPython `pairwise2` module[4] to ensure reproducible and consistent similarity measurements across all protein pairs.

Data Splitting Data splitting was performed based on the connected components of the protein similarity network. Each connected component was assigned entirely to either the training, validation, or test set to avoid overlap between splits and reduce bias from homologous proteins appearing in multiple sets. In our experiments, we used a test set ratio of 0.2 (20% of clusters assigned to the test set), a combined training and validation ratio of 0.8, and within the training and validation set, a training ratio of 0.75 (the remaining 25% used for validation).

Ligand Filtering To prevent chemical-level data leakage and bias, ligands in the training set were compared against all ligands in the test set using Tanimoto similarity computed from Morgan fingerprints. Any training ligand that was highly similar to a test ligand was removed, ensuring that model evaluation reflects generalization to unseen chemical space rather than memorization of structurally similar ligands. In our experiments, a Tanimoto similarity threshold of 0.8 was applied.

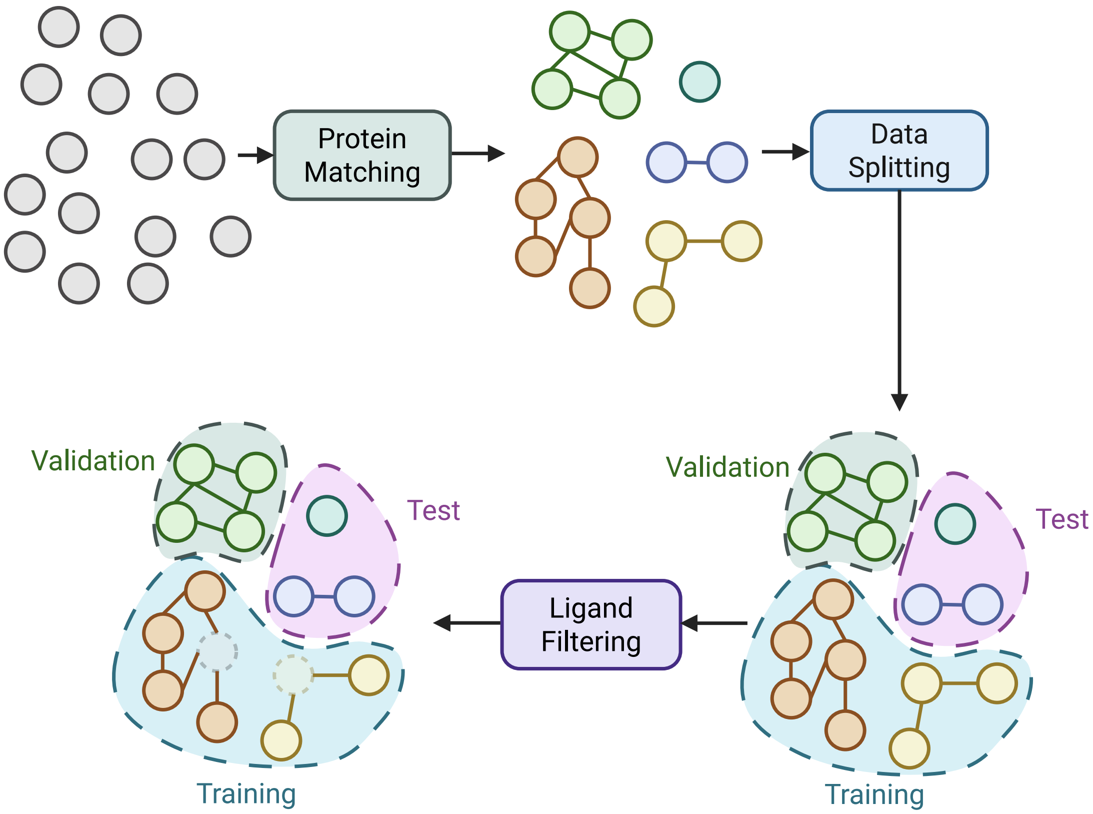

Figure 1: **Overview of the sequence-based protein matching, connected-component data splitting, and ligand similarity filtering procedure.** Each sample consists of a protein sequence and a ligand SMILES. A similarity graph is constructed via **Protein Matching**, and connected components are used for **Data Splitting** into training, validation, and test sets. **Ligand Filtering** is subsequently applied to reduce redundancy between training and test sets, ensuring a rigorous and leakage-aware evaluation.

C Smooth Label Visualization

Figure 2 effectively illustrates the concept of Gaussian smoothing applied to residue-level interaction labels. By converting discrete, binary labels into continuous profiles, it highlights how softening the labels can capture the clustered nature of binding sites and incorporate spatial context beyond rigid cutoffs. The visual comparison clearly demonstrates the impact of kernel width on label diffusion, making it intuitive to understand how smoothing mitigates artifacts and supports more flexible interpretability analyses.

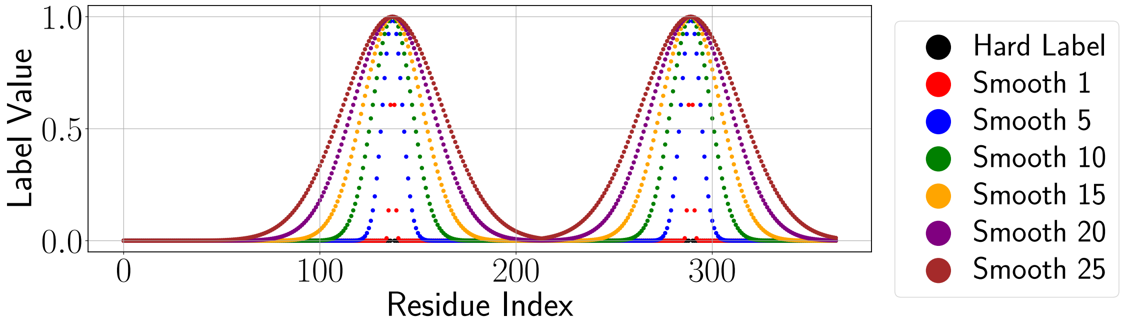

Figure 2: **Gaussian smoothing of interaction labels reflects the continuous nature of binding sites.** An illustration of how discrete, binary interaction labels derived from static crystal structures are transformed into continuous soft-interaction profiles using Gaussian kernel smoothing. Increasing the kernel width creates progressively more diffuse labels that account for the broader spatial context of ligand binding and mitigate artifacts from rigid geometric cutoffs.

D Ablation study on ligand representation

We conducted an end-to-end ablation study on the Davis[5] benchmark to examine whether ligand representations based on chemically meaningful functional groups provide advantages over conventional single-atom representations. Specifically, both the atom-level and functional-group variants were trained directly for binding affinity prediction under the same architecture and optimization protocol, as described in Section F. Performance was evaluated using the metrics defined in Section H. This comparison isolates the effect of ligand granularity and tests whether functional-group-based ligand modeling provides a stronger inductive bias than representing ligands as collections of individual atoms.

Given target embeddings $\mathbf{H}_p \in \mathbb{R}^{R\times D}$ from the Protein Language Model (ESM C) and ligand embeddings $\mathbf{H}_l \in \mathbb{R}^{L\times D}$, where L denotes either the number of atoms or the number of functional groups depending on the ligand representation, the model computes a scalar binding affinity prediction through independent attention pooling over the protein and ligand modalities followed by feature concatenation and regression. The overall architecture used for this end-to-end ablation is illustrated in Figure 3.

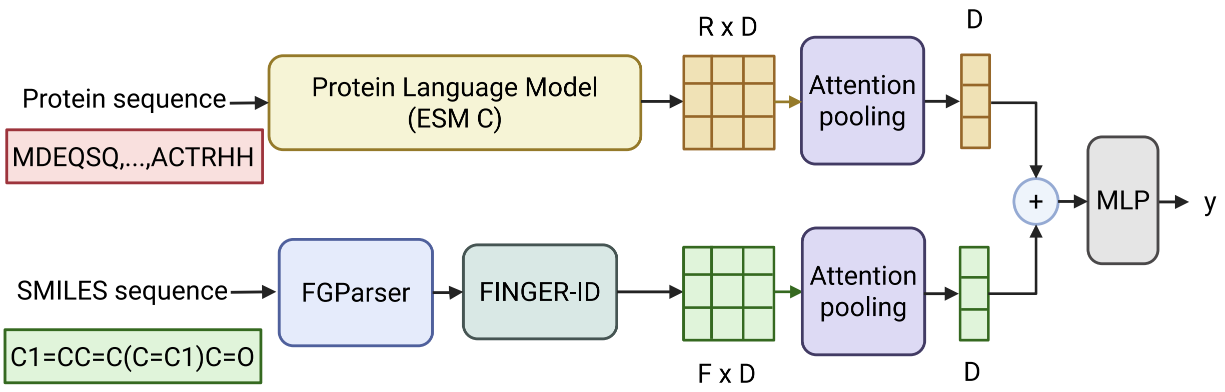

Figure 3: **End-to-end FINGERID-DTA architecture used in the ligand-representation ablation.** Given a protein sequence and a ligand SMILES string, ESM C encodes the protein into residue-level embeddings, while FINGER-ID encodes the ligand into functional-group-level embeddings. The protein and ligand embeddings are independently summarized by attention pooling, concatenated, and passed to an MLP to predict the scalar binding affinity $\hat{y}$. In the atom-level ablation variant, FGParser and the FINGER-ID functional-group encoder are removed and replaced with an atom-level ligand encoder, while the protein encoder, attention pooling layers, and regression head are kept unchanged.

Ligand representation. For the *single-atom* variant, the ligand is represented as a molecular graph whose node embeddings are obtained directly at the atom level. For the *functional-group* variant, the ligand is first decomposed into functional groups and encoded using the FINGER-ID module, producing embeddings at the chemically meaningful substructure level. To assess the effect of encoder depth, we varied the number of GCN layers in the ligand encoder from 3 to 5 and 7 for both representations.

Attention pooling. Given an input sequence embedding matrix $\mathbf{X} \in \mathbb{R}^{L \times D}$, we compute a scalar attention score for each token:

$$e_\ell = f(\mathbf{X}[\ell]) \in \mathbb{R}, \tag{1}$$

where $f(\mathbf{x}_\ell) = \mathbf{w}_2^\top \tanh(\mathbf{W}_1 \mathbf{x}_\ell + \mathbf{b}_1) + b_2$. The attention weights are then obtained by

$$\alpha_\ell = \frac{\exp(e_\ell)}{\sum_{j=1}^{L} \exp(e_j)} \in \mathbb{R}. \tag{2}$$

Finally, the pooled representation is computed as the weighted sum of token embeddings:

$$\mathbf{h}^{\text{pool}} = \sum_{\ell=1}^{L} \alpha_\ell \, \mathbf{X}[\ell] \in \mathbb{R}^D. \tag{3}$$

Final representation and prediction. The pooled protein and ligand embeddings are concatenated and passed to a multilayer perceptron to predict the binding affinity:

$$\mathbf{h} = \left[\mathbf{h}_p^{\text{pool}},\ \mathbf{h}_l^{\text{pool}}\right] \in \mathbb{R}^{2D}, \tag{4}$$

$$\hat{y} = \text{MLP}(\mathbf{h}) \in \mathbb{R}. \tag{5}$$

Results. As shown in Figure 4, the functional-group representation consistently outperforms the atom-level representation across all evaluated GCN depths. The functional-group variant achieves lower MSE and higher CI and r_m^2 at 3, 5, and 7 layers, indicating that the improvement is not tied to a particular depth configuration but instead arises from the ligand representation itself. In addition, the functional-group representation remains more stable as the ligand encoder becomes deeper, whereas the atom-level representation exhibits weaker and more variable performance.

These results suggest that directly modeling ligands as collections of individual atoms may introduce unnecessary granularity for affinity prediction. In contrast, functional groups provide a more compact and chemically organized representation of interaction-relevant ligand features. This supports the use of FINGER-ID as a ligand encoder: it represents ligands at an intermediate chemical scale between coarse molecular fingerprints and atom-level graphs, while preserving context-aware information for each functional group. Unlike fixed fingerprints or global similarity descriptors, FINGER-ID produces embeddings for individual functional groups; unlike purely atom-level graphs, it aggregates local atomic information into chemically meaningful substructures that are more closely aligned with medicinal chemistry interpretation.

To place the Davis benchmark results in the proper methodological context, we summarize

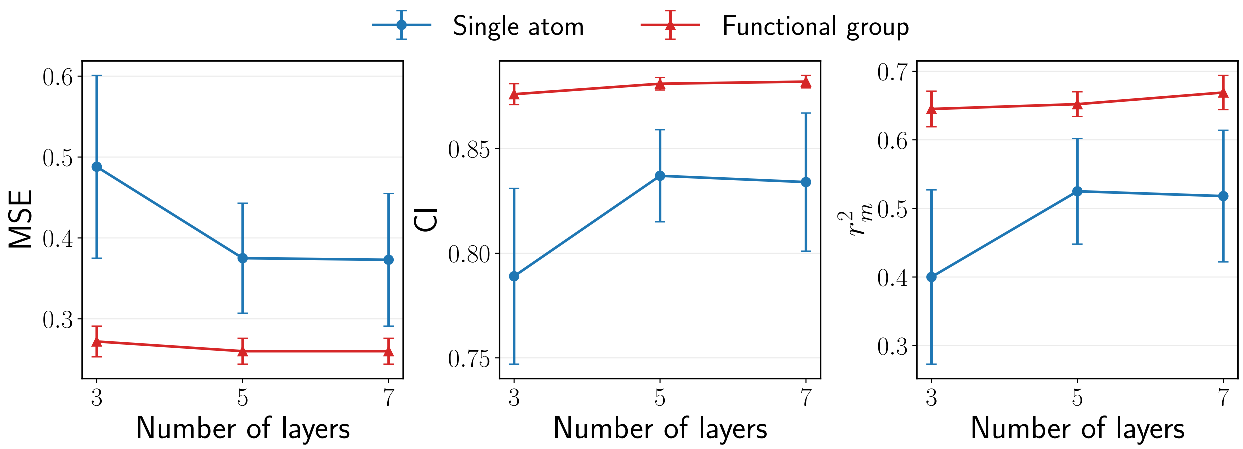

Figure 4: Ablation study comparing single-atom and functional-group ligand representations across different GCN depths on the Davis test set. Performance is reported in terms of MSE, CI, and r_m^2 for models with 3, 5, and 7 GCN layers. Error bars indicate the standard deviation over five independent training splits of the Davis dataset. The functional-group representation consistently outperforms the single-atom representation, yielding lower MSE and higher CI/r_m^2 across all evaluated depths.

the modeling characteristics of the evaluated DTA baselines in Table 1. The compared methods are not homogeneous: they differ in optimization target, ligand granularity, architectural specialization, and the use of auxiliary relational information. Within this landscape, FINGERID-DTA occupies a distinct setting: it does not rely on explicit protein-ligand complex structures or affinity-matrix relational features, but instead evaluates whether a functional-group-level ligand representation can provide a compact, chemically interpretable, and affinity-relevant inductive bias.

Having established that functional-group modeling improves over atom-level ligand encoding, we further evaluate FINGERID-DTA against established DTA baselines on the Davis benchmark in Table 1. FINGERID-DTA outperforms 6 of 14 baselines in MSE, 5 of 14 in CI, and 9 of 14 in r_m^2. These results show that the functional-group-level representation not only improves the internal ablation setting but also retains measurable affinity-prediction utility across classical similarity-based, sequence/attention-based, graph-based, fingerprint-based, and fusion-based DTA methods.

The methods with stronger scalar metrics generally reflect different design choices and,

Table 1: Binding affinity prediction performance on the Davis test set with input characteristics of each method. MSE, CI, and r_m^2 are reported as mean (standard deviation) over five independent training splits of the Davis dataset when available. Arrows indicate the preferred direction for each metric.

**Model**	**Input characteristics**							**Performance**		
	**Prot. Seq.**	**Prot. Struct.**	**Lig. SMILES**	**Lig. Graph**	**FP/ Sim.**	**Aff. Matrix**	**Func. Group**	**MSE** ↓	**CI** ↑	$\mathbf{r_m^2}$ ↑
Classical similarity and feature-based methods										
KronRLS[6]	✓	×	×	×	✓	✓	×	0.379	0.871 (0.001)	0.407 (0.005)
SimBoost[7]	✓	×	×	×	✓	✓	×	0.282	0.872 (0.002)	0.644 (0.006)
Sequence and attention-based neural methods										
DeepDTA[8]	✓	×	✓	×	×	×	×	0.261	0.878 (0.004)	0.630 (0.017)
InceptionDTA(CharVec)[9]	✓	×	✓	×	×	×	×	0.196	0.891 (0.003)	0.625 (0.020)
InceptionDTA(Seq)[9]	✓	×	✓	×	×	×	×	0.242	0.897 (0.002)	0.624 (0.028)
AttentionDTA[10]	✓	×	✓	×	×	×	×	0.216	0.893 (0.005)	0.677 (0.024)
MATT-DTI[11]	✓	×	✓	×	×	×	×	0.227	0.891 (0.003)	0.683 (0.009)
Graph, fingerprint, fusion, and multimodal methods										
GraphDTA[12]	✓	×	×	✓	×	×	×	0.258	0.884 (0.002)	0.656 (0.014)
G-K BertDTA[13]	✓	×	✓	✓	×	×	×	0.267	0.879 (0.003)	0.660 (0.003)
TEFDTA[14]	✓	×	✓	×	✓	×	×	0.264	0.878 (0.002)	0.635 (0.021)
FusionDTA[15]	✓	×	✓	×	×	×	×	0.220	0.903 (0.002)	0.666 (0.008)
BiComp-DTA[16]	✓	×	✓	×	×	×	×	0.237	0.904 (0.001)	0.696 (0.012)
AttentionMGT-DTA[17]	✓	✓	×	✓	×	×	×	0.193	0.891 (0.005)	0.699 (0.027)
DualPG-DTA[18]	✓	✓	✓	✓	×	×	×	**0.169**	**0.908 (0.003)**	**0.778 (0.009)**
Functional-group-level ligand representation										
FINGERID-DTA (7-GCN)	✓	×	✓	✓	×	×	✓	0.254 (0.016)	0.882 (0.003)	0.669 (0.025)

Note. Prot. Seq. denotes protein sequence or sequence-derived representation; Prot. Struct. denotes explicit protein structural input; Lig. Graph denotes atom-level molecular graph input; FP/Sim. denotes molecular fingerprint or ligand/protein similarity features; Aff. Matrix denotes the use of known drug–target affinity or interaction relations as relational input features; Func. Group denotes explicit functional-group-level ligand representation. Checkmarks indicate the presence of a modeling characteristic and should not be interpreted as implying that more checked categories necessarily lead to better affinity prediction performance. Bold values indicate the optimal result for each metric. Values in parentheses denote standard deviations across five independent training splits. Performance results for KronRLS, SimBoost, DeepDTA, AttentionDTA, MATT-DTI, GraphDTA, FusionDTA, and BiComp-DTA were taken from Kalemati et al.[16] The result for DualPG-DTA was taken from Chen et al.[18] Results for the remaining baseline models were obtained from Wu et al.[17] and Kalemati et al.[9]

in some cases, richer input representations or greater task-specific capacity. For instance, DualPG-DTA incorporates pretrained molecular and protein representations, 3D molecular information, graph-based modeling, and dynamic attention mechanisms, while several other high-performing baselines use specialized attention, fusion, or comparison modules tailored for affinity regression. Such components can improve scalar benchmark performance, but they also increase architectural complexity. In contrast, FINGERID-DTA uses a simpler and chemically transparent functional-group-based ligand encoder, without requiring docked protein-ligand poses or explicit complex-level structural inputs. This allows FINGERID-DTA to retain meaningful affinity-prediction performance while preserving interpretability at the

functional-group level.

Taken together, Figure 4 and Table 1 support a consistent conclusion: functional-group-level ligand modeling provides a useful chemical inductive bias for affinity prediction. While highly optimized affinity regressors can improve benchmark scores by increasing architectural complexity or incorporating additional sources of information, FINGERID-DTA achieves meaningful predictive performance with a compact, chemically grounded, and interpretable ligand representation. This highlights FINGER-ID as a practical ligand encoder for linking scalar affinity prediction to functional-group-level chemical reasoning.

E Binding Affinity Predictor

To preserve the learned representations for downstream tasks, the weights of all modules in the LINKER architecture were frozen after training the interaction prediction module, ensuring that no further gradient updates modified the pre-trained parameters.

Given target embeddings $\mathbf{H}_p \in \mathbb{R}^{R\times D}$ from the Protein Language Model (ESM C) and ligand embeddings $\mathbf{H}_l \in \mathbb{R}^{F\times D}$ from FINGER-ID, along with interaction probabilities $\mathbf{P} \in \mathbb{R}^{R\times F\times K}$ from PairwiseUnet, the framework calculates a scalar prediction per protein-ligand complex (Figure 5).

Contact Block This block fuses residue and functional group information using predicted pairwise interaction probabilities. It first computes an edge-strength matrix that quantifies the importance of each residue-functional-group pair. From these strengths, it derives bidirectional attention coefficients and aggregates neighbor information into context vectors, which are then projected and added to the original embeddings to form enriched representations.

- **Edge strength.** Edge strengths aggregate contributions from all interaction types into

a single scalar per residue-functional-group pair:

$$S_{r,f} = \sum_{k=1}^{K} P_{r,f,k}\, w_k, \quad S = [S_{r,f}] \in \mathbb{R}^{R\times F}, \tag{6}$$

where $w_k \in \mathbb{R}$ are learnable importance weights that reweight the predicted interaction-type probabilities.

- **Bidirectional attention.** We convert edge strengths to normalized attention coefficients to capture the asymmetric relevance of each node to its neighbors:

$$\alpha_{r,f}^{p\to l} = \frac{\exp(S_{r,f})}{\sum_{f'=1}^{F}\exp(S_{r,f'})} \in \mathbb{R}, \tag{7}$$

$$\alpha_{r,f}^{l\to p} = \frac{\exp(S_{r,f})}{\sum_{r'=1}^{R}\exp(S_{r',f})} \in \mathbb{R}. \tag{8}$$

where $\alpha_{r,f}^{p\to l}$ measures the importance of the functional group f for residue r, and $\alpha_{r,f}^{l\to p}$ the opposite.

- **Context aggregation & enriched embeddings.** Each residue aggregates a weighted sum of its functional-group neighbors to form a context vector, and each functional group aggregates a weighted sum of its residue neighbors to form its context vector. These context vectors are then projected and added to the original embeddings to produce enriched representations:

$$\mathbf{c}_r^l = \sum_{f=1}^{F} \alpha_{r,f}^{p\to l}\, \mathbf{H}_l[f] \in \mathbb{R}^D, \tag{9}$$

$$\mathbf{c}_f^p = \sum_{r=1}^{R} \alpha_{r,f}^{l\to p}\, \mathbf{H}_p[r] \in \mathbb{R}^D, \tag{10}$$

$$\mathbf{H}'_p[r] = \mathbf{H}_p[r] + \mathrm{proj}_p(\mathbf{c}_r^l) \in \mathbb{R}^D, \tag{11}$$

$$\mathbf{H}'_l[f] = \mathbf{H}_l[f] + \mathrm{proj}_l(\mathbf{c}_f^p) \in \mathbb{R}^D, \tag{12}$$

$$\mathbf{H}'_p = [\mathbf{H}'_p[r]]_{r=1}^{R} \in \mathbb{R}^{R\times D}, \tag{13}$$

$$\mathbf{H}'_l = [\mathbf{H}'_l[f]]_{f=1}^{F} \in \mathbb{R}^{F\times D}. \tag{14}$$

Here, $\mathrm{proj}_p(\cdot)$ and $\mathrm{proj}_l(\cdot)$ denote linear projections (learned) that map the context vectors back to the embedding space before residual addition.

Fusion Block This block summarizes the enriched embeddings into compact pooled vectors and encodes a global signal for each interaction type. The pooled vectors are constructed so that nodes with stronger overall edge connections receive greater importance.

- **Pooling weights and pooled embeddings.** We compute per-node importance scores by summing incident edge strengths, normalize them to obtain pooling weights, and form pooled embeddings as weighted sums of enriched node embeddings:

$$s_r^p = \sum_{f=1}^{F} S_{r,f} \in \mathbb{R}, \quad s_f^l = \sum_{r=1}^{R} S_{r,f} \in \mathbb{R}, \tag{15}$$

$$\beta_r^p = \frac{s_r^p}{\sum_{r'} s_{r'}^p} \in \mathbb{R}, \quad \beta_f^l = \frac{s_f^l}{\sum_{f'} s_{f'}^l} \in \mathbb{R}, \tag{16}$$

$$\mathbf{H}_p^{\mathrm{pool}} = \sum_{r=1}^{R} \beta_r^p\, \mathbf{H}'_p[r] \in \mathbb{R}^D, \quad \mathbf{H}_l^{\mathrm{pool}} = \sum_{f=1}^{F} \beta_f^l\, \mathbf{H}'_l[f] \in \mathbb{R}^D. \tag{17}$$

Intuitively, nodes with larger total edge strength contribute more to the pooled repre-

sentation.

- **Global interaction-type contribution.** A compact global vector records the overall contribution of each interaction type throughout the complex:

$$s_k = \sum_{r=1}^{R} \sum_{f=1}^{F} P_{r,f,k} \, w_k \in \mathbb{R}, \quad \mathbf{s} = [s_1, \ldots, s_K]^\top \in \mathbb{R}^K. \tag{18}$$

which captures the aggregate presence and importance of the interaction type k in the current complex.

Final representation and prediction We obtain the final feature vector by concatenating the pooled target embedding, the global interaction-type vector, and the pooled ligand embedding and then pass it to an MLP to produce the scalar binding affinity prediction.

$$\mathbf{h} = \left[\mathbf{H}_p^{\text{pool}}, \ \mathbf{s}, \ \mathbf{H}_l^{\text{pool}} \right] \in \mathbb{R}^{2D+K}, \tag{19}$$

$$\hat{y} = \text{MLP}(\mathbf{h}) \in \mathbb{R}. \tag{20}$$

F Training Procedure

LINKER training To address the extreme class imbalance inherent in interaction annotations, we adopt the Focal Loss,[19] which dynamically down-weights well-classified examples and emphasizes harder misclassified instances. The Focal Loss is defined as:

$$\mathcal{L}_{\text{focal}} = -\alpha(1 - p_t)^\gamma \log(p_t), \tag{21}$$

where p_t is the predicted probability for the true class, α balances the contribution of positive and negative examples, and γ controls the degree of focus on hard examples. In our

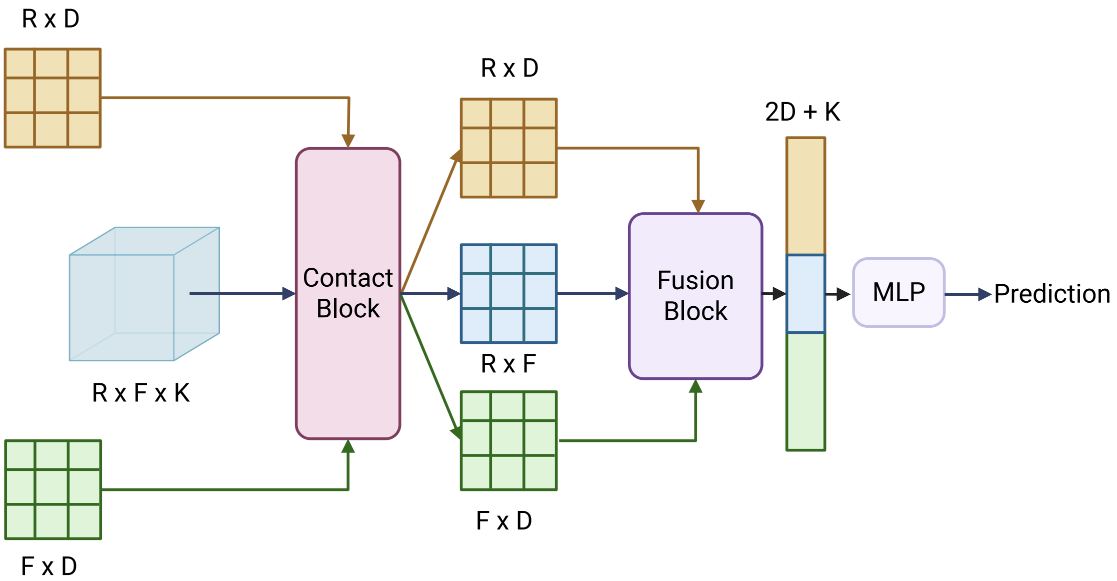

Figure 5: **Architecture of the interaction-aware binding affinity predictor. This framework uses the outputs of the primary LINKER model to predict binding affinity, validating the transferability of the learned representations.** Target embeddings ($\mathbf{H}_p$) from the protein language model and ligand embeddings ($\mathbf{H}_l$) from FINGER-ID are processed alongside the predicted interaction probability tensor (P) from PairwiseUNet. The **Contact Block** fuses these inputs, using the interaction probabilities to weight the aggregation of residue and functional group features, thereby creating contextually enriched embeddings. The subsequent **Fusion Block** pools these enriched representations and incorporates global interaction-type information into a final, fixed-size feature vector. This vector is then passed to a multi-layer perceptron (MLP) that outputs a scalar prediction of binding affinity.

experiments, we set $\alpha = 0.85$ and $\gamma = 1.0$.

Binding Affinity Predictor training For downstream regression, we introduce an interaction-aware neural network that integrates residue embeddings, functional group embeddings, and interaction probabilities produced by LINKER. To train the binding affinity predictor while regularizing the latent space, we optimize a combination of the Mean Squared Error (MSE) loss and the latent alignment loss:

$$\mathcal{L}_{\text{total}} = \mathcal{L}_{\text{MSE}} + \beta\, \mathcal{L}_{\text{latent}}, \tag{22}$$

where β is a hyperparameter controlling the contribution of the latent alignment term.

The MSE loss measures the difference between predicted and true binding affinities:

$$\mathcal{L}_{\text{MSE}} = \frac{1}{N}\sum_{i=1}^{N}(y_i - \hat{y}_i)^2, \tag{23}$$

where y_i and $\hat{y}_i$ denote the ground truth and predicted binding affinities, respectively.

The latent alignment loss combines an InfoNCE term[20] and a uniformity term:[21]

$$\mathcal{L}_{\text{latent}} = \mathcal{L}_{\text{InfoNCE}} + \lambda\, \mathcal{L}_{\text{uniform}}, \tag{24}$$

where λ balances the contribution of the uniformity term.

The latent alignment loss encourages embeddings of samples with similar binding affinities to be close in the latent space while maintaining uniformity in the hypersphere. Let $\mathbf{h}$ denote the final representation defined in 19, and let $\mathbf{h}_i$ be the representation of the i-th sample in a batch. We first normalize each sample embedding to obtain:

$$\mathbf{z}_i = \frac{\mathbf{h}_i}{\|\mathbf{h}_i\|_2} \in \mathbb{R}^{2D+K}. \tag{25}$$

which projects all embeddings onto the unit hypersphere. This normalization removes the influence of vector magnitude and ensures that similarity is determined solely by the angular distance, thereby preventing trivial solutions where embeddings collapse or scale arbitrarily.

The InfoNCE loss encourages embeddings of samples with similar binding affinities to be close to each other in the latent space. For each sample i, we define its positive sample $p(i)$ as the sample in the batch with the most similar binding score (excluding itself). The loss is then computed as:

$$\mathcal{L}_{\text{InfoNCE}} = -\frac{1}{B}\sum_{i=1}^{B}\log\frac{\exp\left(\mathbf{z}_i^\top \mathbf{z}_{p(i)}/\tau\right)}{\sum_{j=1}^{B}\exp\left(\mathbf{z}_i^\top \mathbf{z}_j/\tau\right)}, \tag{26}$$

where τ is a temperature hyperparameter that controls the sharpness of the similarity

distribution.

The Uniformity loss encourages latent vectors to spread on the hypersphere:

$$\mathcal{L}_{\text{uniform}} = \log \frac{1}{B^2} \sum_{i,j=1}^{B} \exp\Big(-2\|\mathbf{z}_i - \mathbf{z}_j\|_2^2 \Big). \tag{27}$$

In our experiments, we set $\beta = 2$, $\lambda = 0.1$, and $\tau = 0.1$.

FINGERID-DTA training FINGERID-DTA was trained end-to-end on the Davis dataset using a composite loss that jointly optimizes point-wise affinity accuracy, pairwise ranking consistency, and r_m^2-based agreement:

$$\mathcal{L}_{\text{total}} = \mathcal{L}_{\text{MSE}} + \lambda_{\text{rank}}\mathcal{L}_{\text{rank}} + \lambda_{r_m^2}\mathcal{L}_{r_m^2}. \tag{28}$$

In our experiments, we set $\lambda_{\text{rank}} = 0.05$ and $\lambda_{r_m^2} = 0.05$.

The MSE loss is defined as:

$$\mathcal{L}_{\text{MSE}} = \frac{1}{N} \sum_{i=1}^{N} (y_i - \hat{y}_i)^2. \tag{29}$$

To encourage correct affinity ordering, we use a pairwise ranking loss over comparable pairs $\mathcal{P} = \{(i, j) \mid |y_i - y_j| > \epsilon\}$:

$$\mathcal{L}_{\text{rank}} = \frac{1}{|\mathcal{P}|} \sum_{(i,j)\in\mathcal{P}} \text{softplus}\left(-\text{sign}(y_i - y_j)(\hat{y}_i - \hat{y}_j)\right). \tag{30}$$

This term penalizes predicted pairwise orderings that disagree with the ground-truth affinity order.

We further include an r_m^2-based differentiable surrogate loss:

$$\mathcal{L}_{r_m^2} = 1 - r^2 \left[1 - (r^2 - r_0^2)^2\right], \tag{31}$$

where r^2 denotes the squared Pearson correlation between $\hat{y}$ and y, and r_0^2 denotes the squared correlation under an origin-constrained regression. This term promotes both correlation and agreement with the ground truth.

Optimization Setup LINKER was trained for 30 epochs and the Binding Affinity Predictor for 80 epochs, both using the Adam optimizer with a learning rate of 2×10^{-5} and batch sizes of 2 and 16, respectively. Validation performance was monitored after each epoch to assess generalization. For the FINGERID-DTA ligand-representation ablation, both atom-level and functional-group variants were trained end-to-end on the Davis dataset for up to 150 epochs with a batch size of 64 and a learning rate of 5×10^{-4}. Early stopping was applied with a patience of 20 epochs based on validation performance. The same protocol was used for both variants to isolate the effect of ligand representation.

G Implementation Details

All experiments were implemented using PyTorch (v2.6.0) with CUDA 12.4 support. GPU-based experiments were conducted on an NVIDIA Tesla P100 GPU equipped with 16 GB of VRAM. CPU-based molecular docking experiments were performed using AutoDock Vina (v1.1.2) on a four-socket system with AMD EPYC 7763 processors, providing 24 GB of memory per CPU. Three-dimensional structural visualizations were generated using UCSF ChimeraX.[22] AlphaFold2 structure predictions were generated using the OpenFold[23] framework.

H Evaluation Metrics

We use the following evaluation metrics in our experiments:

Precision-Recall Curve (PR Curve) The PR curve evaluates the trade-off between precision and recall across varying thresholds:

$$\text{Precision} = \frac{\text{TP}}{\text{TP} + \text{FP}}, \qquad \text{Recall} = \frac{\text{TP}}{\text{TP} + \text{FN}}. \tag{32}$$

The area under the PR curve (PR AUC) summarizes model performance under class imbalance, where positive labels are sparse.

Receiver Operating Characteristic Curve (ROC Curve) The ROC curve plots the true positive rate (TPR) against the false positive rate (FPR):

$$\text{TPR} = \frac{\text{TP}}{\text{TP} + \text{FN}}, \qquad \text{FPR} = \frac{\text{FP}}{\text{FP} + \text{TN}}. \tag{33}$$

The area under the ROC curve (ROC AUC) reflects the model's ability to discriminate between positive and negative classes.

Weighted Precision For soft labels, we compute weighted precision by thresholding the model's attention scores and weighting predictions by the smoothed label values:

$$\text{Weighted Precision} = \frac{\sum_{i=1}^{N} \hat{y}_i \cdot y_i}{\sum_{i=1}^{N} \hat{y}_i}, \tag{34}$$

where $\hat{y}_i \in \{0, 1\}$ is the binary prediction and $y_i \in [0, 1]$ is the smoothed supervision label.

Prevalence Prevalence is simply the proportion of positive samples in the dataset:

$$\text{Prevalence} = \frac{1}{N} \sum_{i=1}^{N} y_i, \tag{35}$$

which corresponds to the horizontal baseline of random predictions in the PR curve.

Enrichment Enrichment measures the improvement of the precision of a model over the prevalence baseline:

$$\text{Enrichment} = \frac{\text{Precision}}{\text{Prevalence}}. \tag{36}$$

High enrichment values indicate that the model retrieves substantially more true positives than expected under random selection, which is particularly informative at low recall in highly imbalanced settings.

Root Mean Squared Error (RMSE) For regression tasks, we use the RMSE to quantify the deviation between predicted and ground-truth values:

$$\text{RMSE} = \sqrt{\frac{1}{N}\sum_{i=1}^{N}\left(\hat{y}_i - y_i\right)^2}, \tag{37}$$

where $\hat{y}_i$ denotes the predicted binding affinity and y_i is the corresponding ground-truth label. Lower RMSE values indicate better predictive accuracy.

Root Mean Square Deviation (RMSD). Root mean square deviation (RMSD) measures the average magnitude of the difference between predicted and ground-truth values. It is defined as:

$$RMSD = \sqrt{\frac{1}{N}\sum_{i=1}^{N}(y_i - \hat{y}_i)^2}, \tag{38}$$

where y_i and $\hat{y}_i$ denote the true and predicted values, respectively, and N is the number of samples. Lower RMSD values indicate better predictive accuracy, with zero corresponding to perfect agreement.

Pearson Correlation Coefficient (Pearson's R) Pearson's correlation coefficient measures the strength of the linear relationship between predicted and ground-truth values. It is

defined as:

$$R = \frac{\sum_{i=1}^{N} (\hat{y}_i - \bar{\hat{y}}) (y_i - \bar{y})}{\sqrt{\sum_{i=1}^{N} (\hat{y}_i - \bar{\hat{y}})^2} \sqrt{\sum_{i=1}^{N} (y_i - \bar{y})^2}}, \tag{39}$$

where y_i and $\hat{y}_i$ denote the true and predicted values, respectively, and $\bar{y}$ and $\bar{\hat{y}}$ are their corresponding means. The coefficient satisfies $R \in [-1, 1]$, where values closer to 1 indicate stronger positive linear agreement, values near 0 indicate weak linear association, and values closer to -1 indicate inverse linear relationship.

Concordance Index (CI) The concordance index (CI) evaluates the ranking consistency between predictions and ground truth, the probability that a randomly chosen comparable pair is ranked in the correct order. It is defined as:

$$\mathrm{CI} = \frac{1}{|\mathcal{P}|} \sum_{(i,j) \in \mathcal{P}} \left(\mathbb{I}(\hat{y}_i > \hat{y}_j) + \frac{1}{2} \mathbb{I}(\hat{y}_i = \hat{y}_j) \right), \tag{40}$$

where $\mathcal{P} = \{(i, j) \mid y_i > y_j\}$ is the set of comparable pairs and $\mathbb{I}(\cdot)$ is the indicator function. CI ranges from 0 to 1, where 1 indicates perfect ranking agreement and 0.5 corresponds to random ranking performance. Higher CI values indicate better ability to preserve the correct ordering of targets, which is particularly useful when relative ranking is more important than absolute error.

Modified Squared Correlation Coefficient (r_m^2) The modified squared correlation coefficient (r_m^2) evaluates both the correlation and agreement between predicted and ground-truth binding affinities. Unlike the standard squared correlation coefficient r^2, which only measures the strength of linear association, r_m^2 penalizes predictions that are highly correlated with the ground truth but systematically shifted away from the ideal regression line. It is defined as:

$$r_m^2 = r^2 \left(1 - \sqrt{r^2 - r_0^2} \right), \tag{41}$$

where r^2 is the squared Pearson correlation coefficient between the predicted values $\hat{y}$ and the ground-truth values y, and r_0^2 is the squared correlation coefficient of the regression line constrained to pass through the origin. Specifically,

$$r^2 = \frac{\left[\sum_{i=1}^{N}(y_i - \bar{y})(\hat{y}_i - \bar{\hat{y}})\right]^2}{\sum_{i=1}^{N}(y_i - \bar{y})^2 \sum_{i=1}^{N}(\hat{y}_i - \bar{\hat{y}})^2}, \tag{42}$$

and

$$r_0^2 = 1 - \frac{\sum_{i=1}^{N}(y_i - k\hat{y}_i)^2}{\sum_{i=1}^{N}(y_i - \bar{y})^2}, \tag{43}$$

where

$$k = \frac{\sum_{i=1}^{N} y_i \hat{y}_i}{\sum_{i=1}^{N} \hat{y}_i^2}. \tag{44}$$

Here, N denotes the number of test samples, y_i and $\hat{y}_i$ denote the true and predicted binding affinities, respectively, and $\bar{y}$ and $\bar{\hat{y}}$ are their corresponding means. Higher r_m^2 values indicate better predictive agreement with the ground truth, with values closer to 1 reflecting stronger correlation and better alignment with the ideal prediction trend.

I Additional Results

I.1 Detailed t-SNE Analysis of All Functional Groups

To investigate the structural integrity and generalizability of the ligand representations, we analyzed high-dimensional embeddings generated by the FINGER-ID module using t-distributed Stochastic Neighbor Embedding (t-SNE).[24] As presented in Figure 6, we visualized the global distribution of all functional groups across the PDBBind and BindingDB datasets, stratified by training, validation, and test partitions.

The resulting projections revealed a stable topological structure across all data splits. The relative spatial arrangement of functional group clusters was preserved from the training set to the held-out test sets, indicating that the FINGER-ID module learns robust, invariant

chemical features rather than memorizing dataset-specific idiosyncrasies. This stability is critical for ensuring that the model generalizes well to unseen ligands in real-world discovery scenarios. Furthermore, the embeddings exhibit a coherent organization governed by chemical similarity. Points in the visualization were colored according to their functional group identifiers, which act as proxies for definitions of chemical substructures. The distinct clustering of similarly colored points confirms the mapping of chemically analogous groups to proximal regions in the latent space. This intrinsic organization persists across both the experimentally derived PDBBind dataset and the computationally docked BindingDB dataset, suggesting that our model captures fundamental physicochemical properties independent of data source or generation method.

Figure 7 focuses on the five most prevalent functional group categories—aromatic compounds, heterocyclic compounds, carboxylic acid derivatives, amines, and halogen derivatives - demonstrating that chemically homogeneous clusters are consistently preserved across training, validation, and test splits in both datasets. This stability highlights that FINGER-ID extracts split-independent and dataset-invariant chemical features, supporting both the interpretability and robustness of the learned representations.

We note that while the broad chemical families form tight primary clusters, there exists fine-grained sub-clustering within specific groups. This dispersion reflects the heterogeneity of chemical environments in which these functional groups reside. Rather than generalizing and collapsing all instances of a functional group into a single point, our model distinguishes them based on their local molecular contexts, such as neighboring atoms and electronic effects. This context-aware embedding strategy enables LINKER to differentiate identical functional groups with varying reactivity and interaction potential, thereby providing a discriminative feature space for precise chemical interaction prediction.

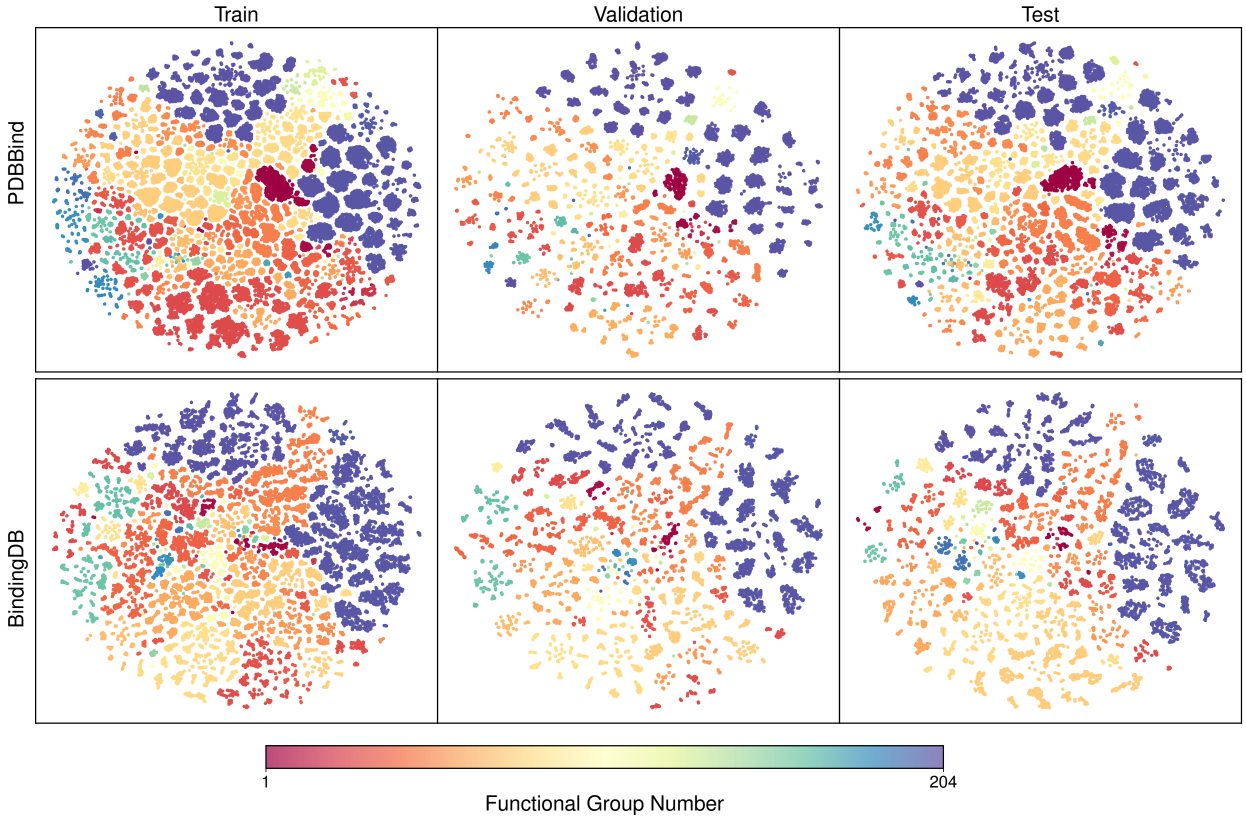

Figure 6: **t-SNE visualization of all functional groups across datasets and splits.** Each point represents a functional group associated with a ligand compound, projected into two-dimensional space using t-SNE based on the FINGER-ID embeddings. Points are colored by functional group number, and the color bar indicates the mapping from FG ID to color. This visualization demonstrates that the relative spatial organization of functional groups is preserved across splits and datasets, highlighting the stability and interpretability of the learned chemical representations.

I.2 Qualitative Examination of Challenging Prediction Cases

To complement the successful qualitative examples presented in the main text, we examine a set of challenging prediction cases in which LINKER does not fully recover the ground-truth residue–functional group interaction patterns. These cases provide insight into the limitations of sequence-based interaction inference while offering a more complete picture of model behavior. Figure 8 presents interaction maps for four protein–ligand complexes (PDB IDs: 1c5t, 2ez7, 3atm, and 4ab9) where predicted hydrogen-bond interactions only partially align with PLIP-derived annotations. In these examples, LINKER captures some key interaction

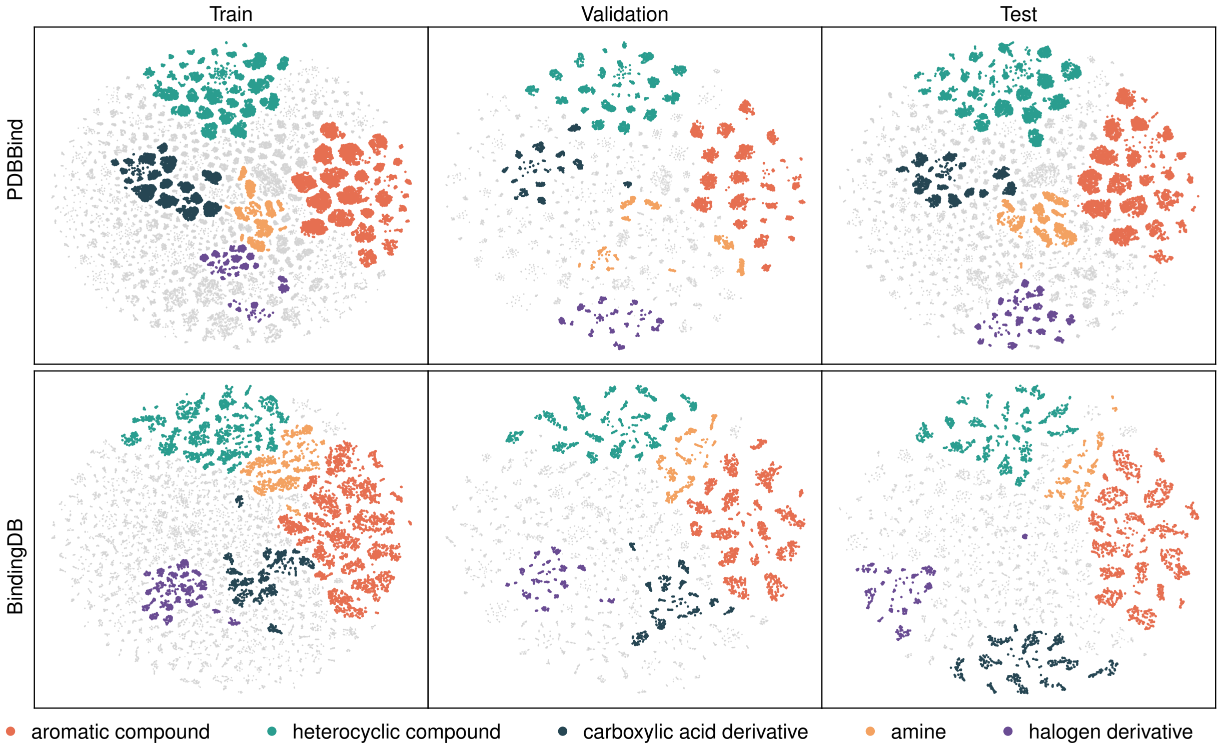

Figure 7: **t-SNE visualization of learned functional group embeddings across datasets and splits.** Scatter plots display two-dimensional predictions of higher-dimension representations generated by the FINGER-ID module for ligand functional groups. Panels are organized by dataset and data partition, and points corresponding to the five most prevalent functional group categories are colored to indicate chemical identity (aromatic compounds, heterocyclic compounds, carboxylic acid derivatives, amines and halogen derivatives). The preservation of distinct, chemically homogeneous clustering across all partitions and datasets demonstrates that the model extracts robust, invariant chemical features that can generalize beyond the specific training examples.

regions but may miss specific contacts or assign confidence to nearby but incorrect residue positions. Such discrepancies are most pronounced in systems with sparse interaction labels or subtle geometric constraints, underscoring the inherent difficulty of inferring fine-grained interaction details from sequence information alone.

In the multi-residue interaction cases shown in Figure 9 (PDB IDs: 1c5t and 2ez7), the ground-truth annotations involve multiple interacting residues distributed across the binding site, leading to overlapping or competing interaction modes and more diffuse predicted interaction hotspots along the protein sequence. Similarly, in the ligand-dependent cases

shown in Figure 10 (PDB IDs: 3atm and 4ab9), LINKER struggles to consistently adapt its predictions when the same protein interacts with chemically distinct ligands, highlighting limitations in capturing subtle ligand-induced binding site reorganization.

Importantly, despite these inaccuracies at the interaction-type or residue-level resolution, LINKER consistently identifies the correct binding pocket regions along the protein sequence across all challenging cases. In each example, elevated prediction scores remain concentrated around the true ligand-binding site, even when individual interactions are misclassified or incompletely recovered. This behavior suggests that while precise interaction typing remains challenging in certain regimes, the model retains a robust ability to localize ligand-binding regions from sequence alone. As such, LINKER may still provide valuable guidance for binding site identification and downstream analysis, even in cases where detailed interaction prediction is imperfect. Together, these challenging cases help delineate the current boundaries of LINKER's capabilities and motivate future improvements, including richer ligand representations, enhanced modeling of complex interaction chemistries, and tighter integration of contextual structural information.

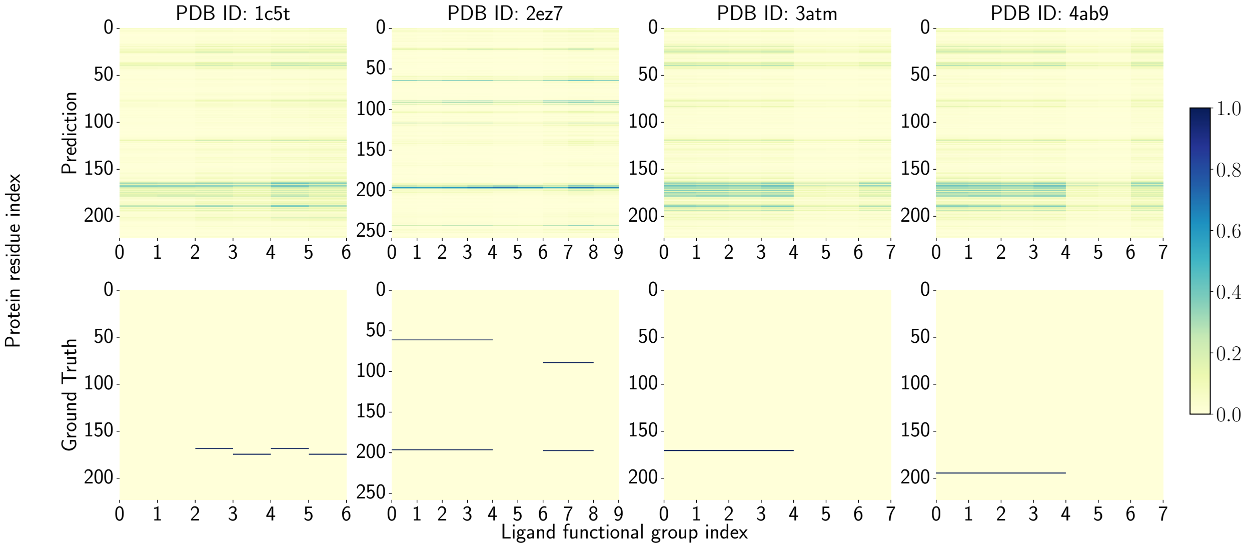

Figure 8: **Challenging prediction cases where predicted interaction maps show partial alignment with ground-truth annotations.** Qualitative comparison between LINKER-predicted residue–functional group interaction maps and PLIP-derived ground-truth annotations for hydrogen bonds across four protein–ligand complexes. The heatmaps plot interaction probabilities between protein residue indices (y-axis) and ligand functional group indices (x-axis). These cases exhibit partial alignment, missed contacts, or mislocalized interaction regions relative to the ground truth, highlighting limitations of sequence-based inference in capturing complex binding interactions.

References

(1) Jain, A. N. Surflex: fully automatic flexible molecular docking using a molecular similarity-based search engine. *Journal of medicinal chemistry* **2003**, *46*, 499–511.

(2) Needleman, S. B.; Wunsch, C. D. A general method applicable to the search for similarities in the amino acid sequence of two proteins. *Journal of molecular biology* **1970**, *48*, 443–453.

(3) Henikoff, S.; Henikoff, J. G. Amino acid substitution matrices from protein blocks. *Proceedings of the National Academy of Sciences* **1992**, *89*, 10915–10919.

(4) Cock, P. J.; Antao, T.; Chang, J. T.; Chapman, B. A.; Cox, C. J.; Dalke, A.; Friedberg, I.; Hamelryck, T.; Kauff, F.; Wilczynski, B.; others Biopython: freely available Python

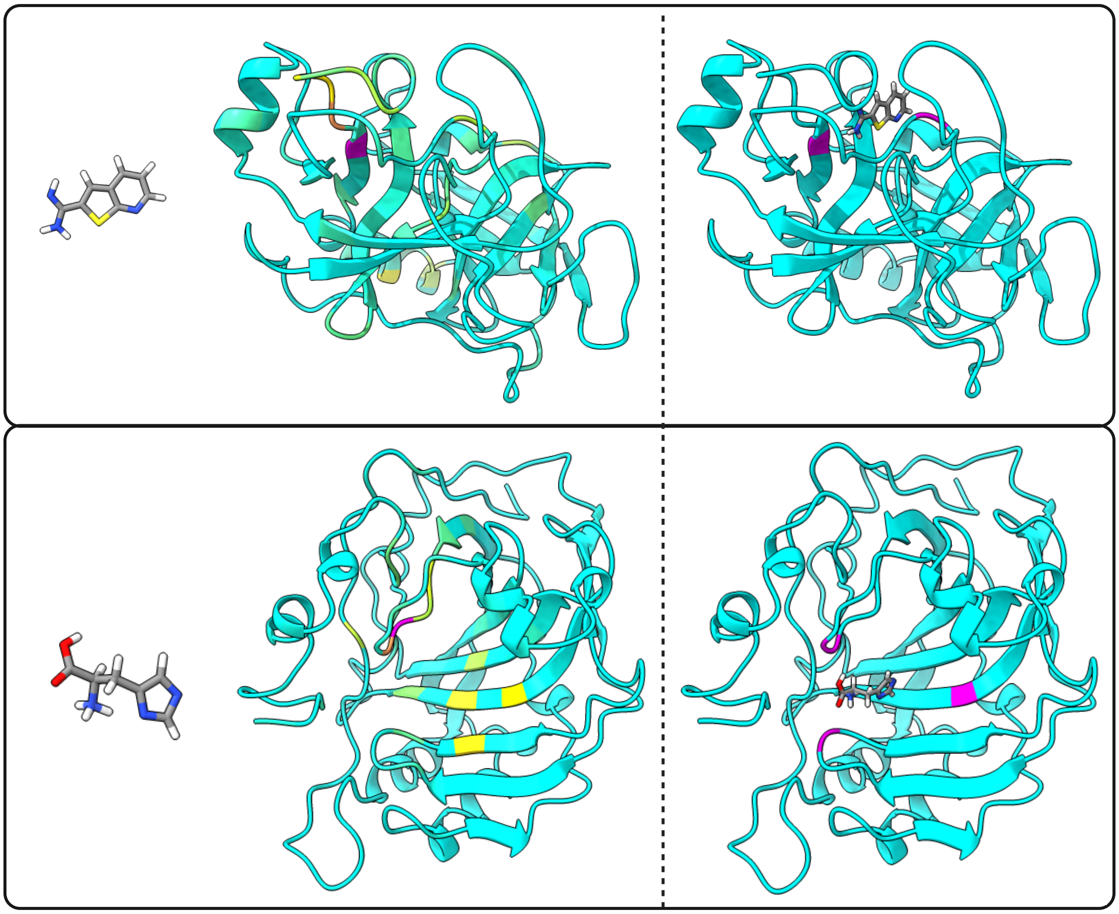

Figure 9: **Challenging prediction cases involving complex multi-residue interaction environments.** From top to bottom, the protein–ligand complexes correspond to PDB IDs 1c5t and 2ez7, respectively. From left to right, the ligand structure, the predicted binding probability aggregated across all ligand functional groups along the protein sequence, and the corresponding protein–ligand complex are shown. Predicted binding probabilities range from 0 to 1 and are color-coded from cyan to yellow to magenta. In these cases, multiple interacting residues are annotated within the binding site, leading to overlapping or competing interaction modes and more diffuse predicted interaction hotspots along the protein sequence.

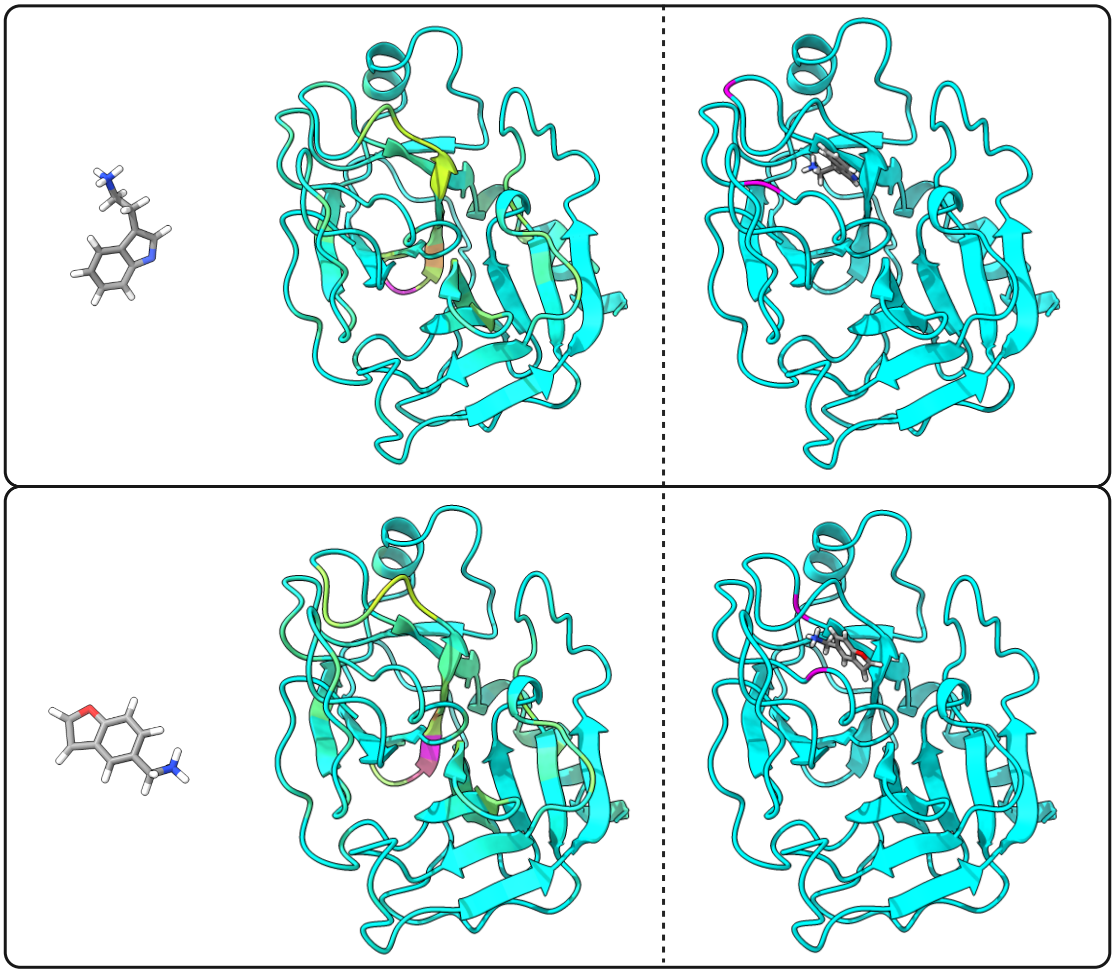

Figure 10: **Challenging prediction cases for ligand-dependent interaction patterns of the same protein.** From top to bottom, the protein–ligand complexes correspond to PDB IDs 3atm and 4ab9, respectively. From left to right, the ligand structure, the predicted binding probability aggregated across all ligand functional groups along the protein sequence, and the corresponding protein–ligand complex are shown. Predicted binding probabilities range from 0 to 1 and are color-coded from cyan to yellow to magenta. These cases highlight scenarios where LINKER struggles to consistently adapt predictions to ligand-specific binding patterns using sequence-level cues alone.

tools for computational molecular biology and bioinformatics. *Bioinformatics* **2009**, *25*, 1422–1423.

(5) Davis, M. I.; Hunt, J. P.; Herrgard, S.; Ciceri, P.; Wodicka, L. M.; Pallares, G.; Hocker, M.; Treiber, D. K.; Zarrinkar, P. P. Comprehensive analysis of kinase inhibitor selectivity. *Nature biotechnology* **2011**, *29*, 1046–1051.

(6) Pahikkala, T.; Airola, A.; Pietilä, S.; Shakyawar, S.; Szwajda, A.; Tang, J.; Aittokallio, T. Toward more realistic drug–target interaction predictions. *Briefings in bioinformatics* **2015**, *16*, 325–337.

(7) He, T.; Heidemeyer, M.; Ban, F.; Cherkasov, A.; Ester, M. SimBoost: a read-across approach for predicting drug–target binding affinities using gradient boosting machines. *Journal of cheminformatics* **2017**, *9*, 24.

(8) Öztürk, H.; Özgür, A.; Ozkirimli, E. DeepDTA: deep drug–target binding affinity prediction. *Bioinformatics* **2018**, *34*, i821–i829.

(9) Kalemati, M.; Emani, M. Z.; Koohi, S. InceptionDTA: Predicting drug-target binding affinity with biological context features and inception networks. *Heliyon* **2025**, *11*.

(10) Zhao, Q.; Duan, G.; Yang, M.; Cheng, Z.; Li, Y.; Wang, J. AttentionDTA: Drug–target binding affinity prediction by sequence-based deep learning with attention mechanism. *IEEE/ACM transactions on computational biology and bioinformatics* **2022**, *20*, 852–863.

(11) Zeng, Y.; Chen, X.; Luo, Y.; Li, X.; Peng, D. Deep drug-target binding affinity prediction with multiple attention blocks. *Briefings in bioinformatics* **2021**, *22*, bbab117.

(12) Nguyen, T.; Le, H.; Quinn, T. P.; Nguyen, T.; Le, T. D.; Venkatesh, S. GraphDTA: predicting drug–target binding affinity with graph neural networks. *Bioinformatics* **2021**, *37*, 1140–1147.

(13) Qiu, X.; Wang, H.; Tan, X.; Fang, Z. GK BertDTA: a graph representation learning and semantic embedding-based framework for drug-target affinity prediction. *Computers in Biology and Medicine* **2024**, *173*, 108376.

(14) Li, Z.; Ren, P.; Yang, H.; Zheng, J.; Bai, F. TEFDTA: a transformer encoder and fingerprint representation combined prediction method for bonded and non-bonded drug–target affinities. *Bioinformatics* **2024**, *40*, btad778.

(15) Yuan, W.; Chen, G.; Chen, C. Y.-C. FusionDTA: attention-based feature polymerizer and knowledge distillation for drug-target binding affinity prediction. *Briefings in Bioinformatics* **2022**, *23*, bbab506.

(16) Kalemati, M.; Zamani Emani, M.; Koohi, S. BiComp-DTA: Drug-target binding affinity prediction through complementary biological-related and compression-based featurization approach. *PLOS Computational Biology* **2023**, *19*, e1011036.

(17) Wu, H.; Liu, J.; Jiang, T.; Zou, Q.; Qi, S.; Cui, Z.; Tiwari, P.; Ding, Y. AttentionMGT-DTA: A multi-modal drug-target affinity prediction using graph transformer and attention mechanism. *Neural Networks* **2024**, *169*, 623–636.

(18) Chen, Y.; Huang, J.; Liu, C.; Zhang, S.; Li, X.; Zhang, Z.; Chen, T.-G.; Wang, L. DualPG-DTA: A Large Language Model-Powered Graph Neural Network Framework for Enhanced Drug-Target Affinity Prediction and Discovery of Novel CDK9 Inhibitors Exhibiting in Vivo Anti-Leukemia Activity. *Advanced Science* **2026**, e13099.

(19) Lin, T.-Y.; Goyal, P.; Girshick, R.; He, K.; Dollár, P. Focal loss for dense object detection. Proceedings of the IEEE international conference on computer vision. 2017; pp 2980–2988.

(20) Oord, A. v. d.; Li, Y.; Vinyals, O. Representation learning with contrastive predictive coding. *arXiv preprint arXiv:1807.03748* **2018**,

(21) Wang, T.; Isola, P. Understanding contrastive representation learning through alignment and uniformity on the hypersphere. International conference on machine learning. 2020; pp 9929–9939.

(22) Pettersen, E. F.; Goddard, T. D.; Huang, C. C.; Meng, E. C.; Couch, G. S.; Croll, T. I.; Morris, J. H.; Ferrin, T. E. UCSF ChimeraX: Structure visualization for researchers, educators, and developers. *Protein science* **2021**, *30*, 70–82.

(23) Ahdritz, G.; Bouatta, N.; Floristean, C.; Kadyan, S.; Xia, Q.; Gerecke, W.; O'Donnell, T. J.; Berenberg, D.; Fisk, I.; Zanichelli, N.; others OpenFold: retraining AlphaFold2 yields new insights into its learning mechanisms and capacity for generalization. *Nature methods* **2024**, *21*, 1514–1524.

(24) Maaten, L. v. d.; Hinton, G. Visualizing data using t-SNE. *Journal of machine learning research* **2008**, *9*, 2579–2605.
